# Hafnium vs. Zirconium, the Perpetual Battle for Supremacy in Catalytic Olefin Polymerization: A Simple Matter of Electrophilicity?

**DOI:** 10.3390/polym13162621

**Published:** 2021-08-06

**Authors:** Antonio Vittoria, Georgy P. Goryunov, Vyatcheslav V. Izmer, Dmitry S. Kononovich, Oleg V. Samsonov, Francesco Zaccaria, Gaia Urciuoli, Peter H. M. Budzelaar, Vincenzo Busico, Alexander Z. Voskoboynikov, Dmitry V. Uborsky, Christian Ehm, Roberta Cipullo

**Affiliations:** 1 Dipartimento di Scienze Chimiche, Università di Napoli Federico II, Via Cintia, 80126 Napoli, Italy; antonio.vittoria@unina.it (A.V.); gaia.urciuoli@unina.it (G.U.); p.budzelaar@unina.it (P.H.M.B.); busico@unina.it (V.B.); 2 Department of Chemistry, Lomonosov Moscow State University, 1/3 Leninskie Gory, 119991 Moscow, Russia; goryunov@org.chem.msu.ru (G.P.G.); izmer_slava@mail.ru (V.V.I.); dkononovich@org.chem.msu.ru (D.S.K.); oleg.samson@mail.ru (O.V.S.); voskoboy@med.chem.msu.ru (A.Z.V.); 3 Dipartimento di Chimica, Biologia e Biotecnologie and CIRCC, Universitaà di Perugia, 06123 Perugia, Italy; francesco.zaccaria@unipg.it

**Keywords:** hafnocenes, olefin polymerization, iPP, molecular catalysts, QSAR, high-temperature performance

## Abstract

The performance of *C*_2_-symmetric *ansa*-hafnocene catalysts for isotactic polypropylene typically deteriorates at increasing temperature much faster than that of their zirconium analogues. Herein, we analyze in detail a set of five Hf/Zr metallocene pairs—including some of the latest generation catalysts—at medium- to high-polymerization temperature. Quantitative structure–activity relationship (QSAR) models for stereoselectivity, the ratio allyl/vinyl chain ends, and 2,1/3,1 misinsertions in the polymer indicate a strong dependence of polymerization performance on electrophilicity of the catalyst, which is a function of the ligand framework and the metal center. Based on this insight, the stronger performance decline of hafnocenes is ascribed to electrophilicity-dependent stabilization effects.

## 1. Introduction

Systems active in olefin polymerization have been reported for most transition metals, but large-scale industrial applications are essentially limited to Cr (Phillips-type catalysts) [1] and group 4 metals (Ti: heterogeneous Ziegler–Natta systems; Ti, Zr, or Hf: homogenous molecular catalysts) [2,3,4]. The latter are employed in a wide range of applications, from high-density polyethylene (HDPE) to linear, low-density polyethylene (LLDPE), to ethylene propylene diene monomer (EPDM) rubber, to isotactic polypropylene (iPP) production [5]. The central role of group 4 metals in the industrial production of polyolefin resins is largely a combination of two factors: affordable metal price and high activity of the resulting catalysts.

While titanium-based hemi-metallocene complexes have been successfully used for high-temperature solution polymerization [3,4], Ti-based metallocenes are virtually of zero commercial importance due to their high tendency to rapidly deactivate under typical polymerization conditions [6,7,8]. Zirconium and hafnium, which share very similar properties (e.g., ionic radii) due to the lanthanide contraction [9,10], have puzzled olefin polymerization chemists for quite some time [11,12].

When comparing pairs of Hf and Zr catalysts, some general and characteristic differences emerge. For instance, hafnocenes have long been considered intrinsically less active than the corresponding zirconocenes, but it has been demonstrated that this is mostly due to the reactions or pre-equilibria with the aluminum-based cocatalyst [13]. Conversely, the higher regioselectivity and molecular weight capability of the hafnocenes compared with zirconocenes are more difficult to rationalize and often explained in terms of the so-called “hafnium effect” [14,15]. Interestingly, although the difference between Zr–C and Hf–C bond lengths is small, the latter are shorter and one would, therefore, expect also slightly tighter active pockets and, correspondingly, higher stereoselectivity for Hf. This has been, indeed, observed for some Hf-based post-metallocene catalysts of the bis(phenolate) type [16,17], while it is generally not the case for hafnocenes at industrially relevant high temperatures [15].

**Figure 1:**
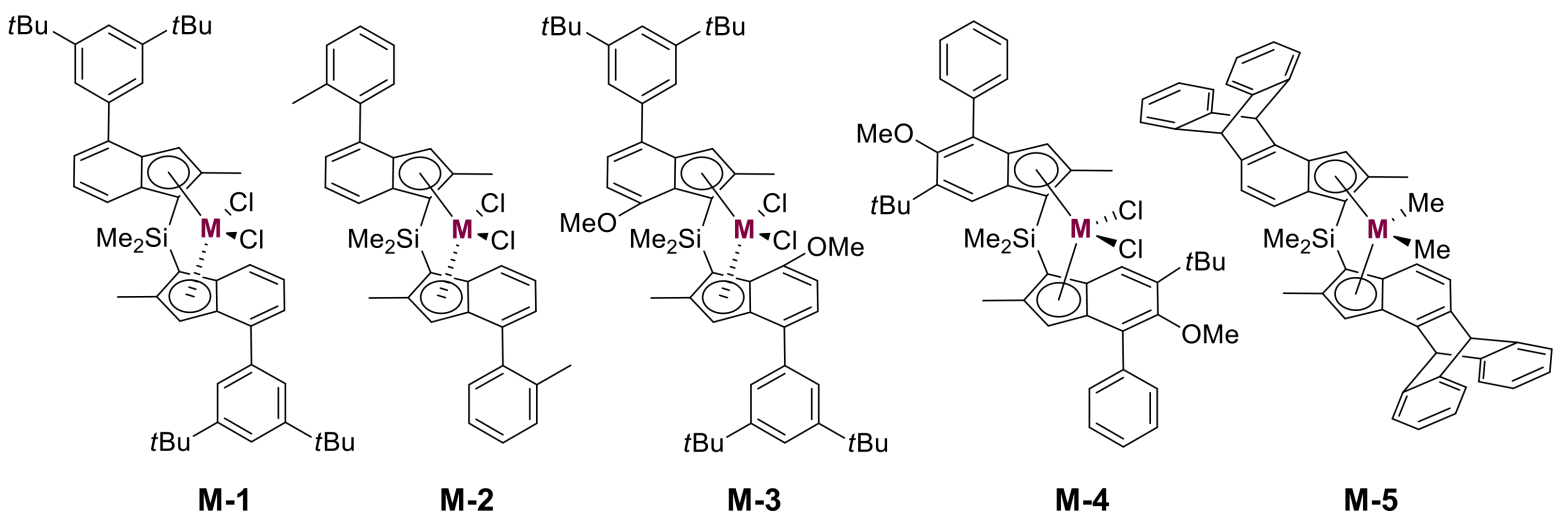
Precatalyst pairs **M-1 to M-5** (M = Zr or Hf) screened in propene polymerization at *T*_p_ = 60 and 100 °C.

As a matter of fact, the only metallocene catalyst known to produce perfect iPP (at 0 °C) is Hf-based (**Hf-3** in Figure 1) [18]. However, for high-temperature applications, Zr-based metallocenes actually appear to outperform the Hf derivatives, as underlined by some leading catalysts for iPP developed by industry like ExxonMobil’s *rac*-Me_2_Si(2-methyl-4-carbazolyl)_2_ZrCl_2_ and Borealis’ *rac*-Me_2_Si(2-methyl-4-(3,5-*tert*-butyl)phenyl-7-methoxy)_2_ZrCl_2_ (**Zr-4** in Figure 1) [19,20,21].

This is because the typical performance decline of metallocenes at higher polymerization temperature appears more marked for hafnocenes than for zirconocenes. Importantly, this decline is likely also a function of the ancillary ligand backbone, although this dependence has been poorly explored in the literature.

Among the various interpretations proposed, the group of Rieger recently attributed the “hafnium effect” to the different covalent vs. ionic character and, therefore, strength of the Zr–C vs. Hf–C bonds [15]. Nevertheless, a conclusive explanation, especially regarding the steep Hf performance decline, is still pending.

The present paper aims to contribute to understanding the origin of the “hafnium effect” by comparing the performances of some zirconocenes (**Zr-1** to **Zr-5**) with those of their corresponding hafnocenes (**Hf-1** to **Hf-5**, Figure 1) in propene polymerization at medium (60 °C) to high (100 °C) reaction temperature. Testing both Hf and Zr derivatives is commonplace in polyolefin R&D and predicting the point where a Zr-based catalyst starts to outperform its Hf analogue could contribute to a streamlined R&D approach.

## 2. Materials and Methods

### 2.1. Catalyst Synthesis

The ancillary ligand precursors bis[4 -(3,5-di-*tert*-butylphenyl)-2-methyl-1*H*-inden-1-yl](dimethyl)silane [22], bis[4-(2-methylphenyl)-2-methyl-1*H*-inden-1-yl](dimethyl)silane [23], bis[4-(3,5-di-*tert*-butylphenyl)-7-methoxy-2-methyl-1*H*-inden-1-yl](dimethyl)silane [18], bis(6-*tert*-butyl-5-methoxy-2-methyl-4-phenyl-1*H*-inden-1-yl)(dimethyl)silane [24], and bis (2-methyl-1H-cyclopenta[a]triptycene-1-yl)(dimethyl)silane [24], as well as the zirconocenes **Zr-1** to **Zr-5** [23,24,25], were synthesized according to published procedures. The synthesis of **Hf-1** to **Hf-5** is detailed in the Supporting information.

### 2.2. Polymer Synthesis and Characterization

All polymerization experiments were performed in a Freeslate Parallel Pressure Reactor setup with 48 reaction cells (PPR48), fully contained in a triple MBraun glovebox under nitrogen. The cells (5.0 mL working volume) feature 800-rpm magnetically coupled stirring and individual online reading/control of temperature, pressure, monomer uptake, and uptake rate. The setup and the operating protocol are described in full detail in [26,27] and the Supporting Information, and they have been used successfully in various homogenous and heterogeneous polymerization studies [16,23,25,28,29,30,31,32,33]. Polymerization conditions are detailed in the Supplementary Materials Table S1. The catalysts were not pre-activated prior to injection into the PPR cells. All experiments were performed at least in duplicate. Monomer was fed on demand.

The polymers were characterized by (1) high-temperature GPC with a Freeslate Rapid-GPC setup; (2) quantitative ^13^C NMR with a Bruker Avance III 400 spectrometer equipped with a high-temperature cryoprobe (for 5-mm OD tubes) and a pre-heated robotic sample changer; and (3) DSC with a Mettler Toledo DSC-822 calorimeter. Polymer melting points (*T*_m_) were collected from the second heating scan. All results are averages on polymer samples produced in polymerization experiments performed at least in duplicate. More details can be found in the Supplementary Materials Table S2 and Figures S1–S4.

### 2.3. Computational Details

#### 2.3.1. Precursor Structures for QSAR Models

Following the protocol proposed in [34], dichloride metallocenes were fully optimized using the Gaussian 16 software package (Gaussian 16, Revision A.1, Gaussian, Inc., Wallingford, CT, USA) [35], in combination with the OPTIMIZE routine of Baker [36,37] and the BOpt software package [38], at the TPSSTPSS [39]/cc-pVDZ(-PP) [40,41,42] level of theory, using a small core pseudo-potential on Zr [43,44]. The protocol has been successfully used, in combination with M06-2X [45] single-point energy (SP) corrections, to address several polymerization-related problems: absolute barrier heights for propagation [46], comonomer reactivity ratios [47,48,49], metal–carbon bond strengths under polymerization conditions [50,51,52,53], electronic and steric tuning of molar mass capability [54], and quantitative structure–activity relationship (QSAR) modeling [23,25,31,32,33]. The density-fitting approximation (Resolution of Identity, RI) [55,56,57,58] and standard Gaussian16 quality settings were used at the optimization stage and SP calculations. All structures represent true minima (as indicated by the absence of imaginary frequencies). Buried volume descriptors were calculated using the SambVca 2.0 program [59]. NPA charges were determined from SP calculations at the M06-2X/cc-pVTZ(-PP) level of theory using the NBO 3.1 program [60], implemented in Gaussian 16.

#### 2.3.2. PES Calculations for Catalyst Pair **M-2**

For the free energy profile for chain end epimerization in Figure 5, all structures were optimized at the MN15 [61] (SMD [62])/cc-pVTZ-(PP)//MN15/cc-pVDZ-(PP) level of theory using Gaussian 16 and the density-fitting approximation and represent either minima (as indicated by the absence of imaginary frequencies) or transition states (as indicated by one imaginary frequency corresponding to the reaction coordinate). MN15 was chosen as the barrier height for β-H elimination (BHE) from a tertiary alkyl is very functional dependent. Dispersion corrections at the optimization level appear crucial to reproduce the necessary close energetic similarity of BHE(secondary alkyl)/BHE(tertiary alkyl)/BME, as seen in the experimental ratio of chain epimerization (via a sequence of BHE events) to chain transfer (via BME or BHE_T_, see main text for definitions).

## 3. Results and Discussion

### 3.1. Polymerization Screening

The catalysts **Hf-1** to **Hf-5** were screened in propene homopolymerization on a PPR48 platform at 60 and 100 °C, and the polymer samples were characterized by ^13^C NMR spectroscopy for stereo- and regioerror quantification and by GPC for molecular weight determination (Table 1). Data for the Zr derivatives **Zr-1** to **Zr-5**, generated under identical polymerization conditions, are provided for comparison, and taken from references [23,24,25,32].

At *T*_p_ = 60 °C, the regioselectivity of the Hf derivatives is consistently better than that of the Zr analogues, and, except for **M-3** and **M-5**, Hf shows a higher tendency to isomerize 2,1 units to 3,1 units. On the other hand, the stereoselectivity of all hafnocenes is consistently much worse (up to 6-fold more stereoerrors) compared to the Zr analogues. Regarding molar mass capability, no clear trend is observed favoring either Zr or Hf. **Zr-1** and **Zr-2** surpass their respective Hf counterparts, while **Zr-3** has a 4-fold lower molar mass capability than **Hf-3** (400 kDa vs. 1.5 MDa). The **M-4** and **M-5** pairs show very similar molar mass capability.

As expected, at *T*_p_ = 100 °C, catalytic performance worsens for all catalysts. Compared with the zirconocenes, the Hf catalysts still show the higher regioselectivity, with a higher tendency to rearrange the 2,1 units into 3,1 units, but their stereoselectivity is considerably lower. Molar mass capability is worse for all Hf catalysts, except for **Hf-3**; however, even **Hf-3** loses much of its advantage over **Zr-3** (from 4-fold higher *M*_n_ at 60 °C to less than 2-fold at 100 °C). On average, molar mass capability of the Zr-based catalysts decreases by a factor of 8–10. The performance of the Hf-based metallocenes decreases faster (13- to 25-fold).

**Scheme 1:**
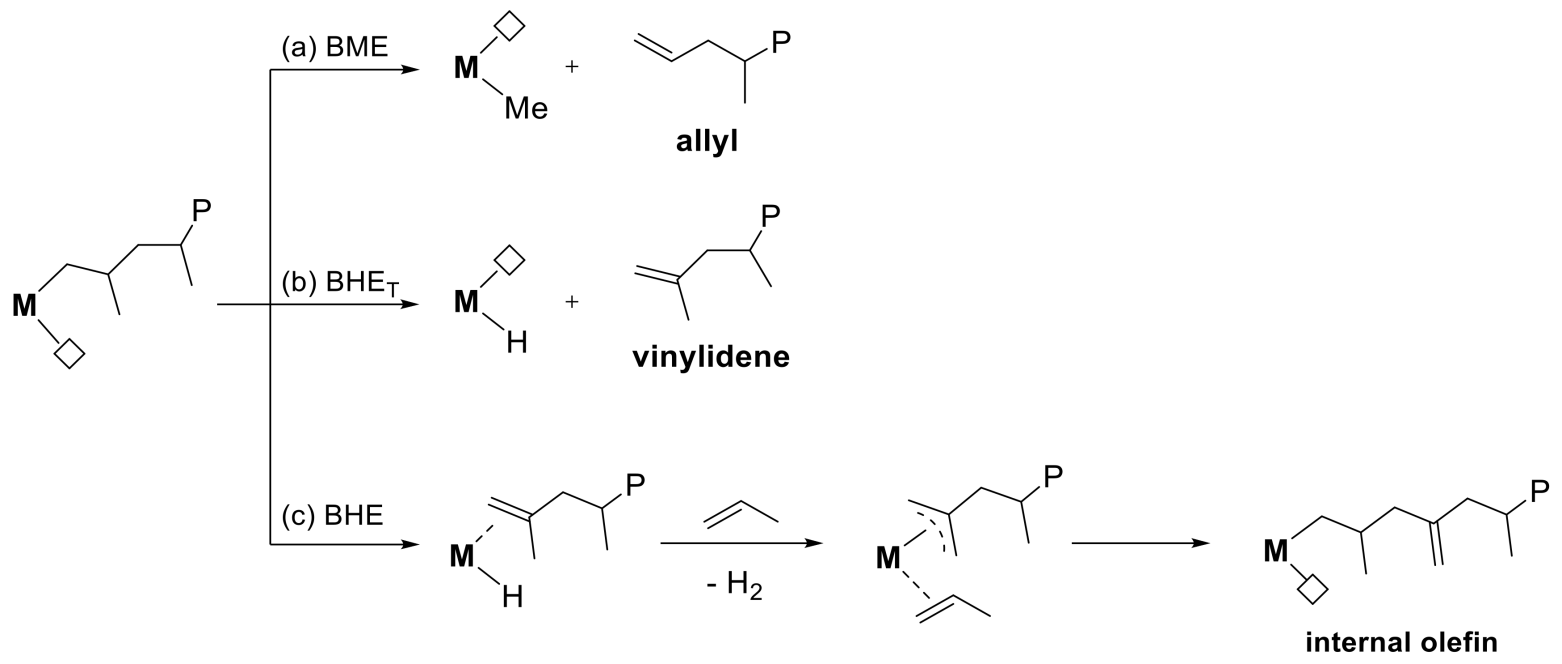
Elimination events leading to chain transfer or in-chain functionalities: (**a**) chain transfer via BME, (**b**) chain transfer via BHE_T_, (**c**) formation of internal olefin via BHE and subsequent C–H activation. The representative case of a M–polymeryl chain after 1,2 insertion is considered here [63,64].

The strong decrease in molar mass capability going to *T*_p_ =100 °C deserves a more detailed analysis. Table 2 reports the results of the chain end analysis performed by ^1^H NMR spectroscopy on polymer samples obtained at 100 °C. With some exceptions, the dominating chain transfer pathway is BME, leading to the formation of allyl chain ends (Scheme 1a). BHE events leading to chain transfer (BHE_T_) are the other chain release pathway generating vinylidene chain end groups (Scheme 1b). Internal olefins, originating from BHE and subsequent allylic C−H bond activation (Scheme 1c), do not contribute to the molecular weight drop. It is important to note that BME is the leading chain transfer event for all hafnocenes, resulting in 66–93% allyl-terminated iPP chains. On the contrary, for the zirconocenes in the catalyst set, BME contributes 17–65% of all chain transfer events at *T*_p_ = 100 °C, with **Zr-3** showing the lowest contribution.

It should be emphasized that molar masses estimated by GPC are nearly identical to those estimated by NMR spectroscopy, implying that chain transfer reactions to aluminum, generating saturated chain ends [11,63], are negligible under the applied conditions. As a matter of fact, polymerizations performed in the presence and absence of 2,6-di-*tert*-butyl-4-methylphenol (BHT), a well-established scavenger for Al-trialkyls [65,66,67], yield polymers with identical *M*_n_.

### 3.2. Differences between Hf and Zr

#### 3.2.1. Can Zirconocene QSAR Models Be Easily Extended to Hf?

Although QSAR modeling is essentially a statistical approach, QSAR models using interpretable descriptors can offer mechanistic insights [68]. The performance of *ansa-*hafnocenes **Hf-1** to **Hf-5** was first tentatively analyzed by applying recently developed QSAR models for stereoselectivity, regioselectivity, and molar mass capability of *ansa*-zirconocenes at 60 °C [23,25]. These models are mathematical combinations of simple and chemically meaningful descriptors selected from a set of several 3D-steric descriptors, determined using Cavallo’s SambVca 2.0 program that maps the distribution of steric bulk in the active pocket [59], and one electronic descriptor.

Using these models to predict the performance of hafnocenes at 60 °C proved futile: The deviations from observed experimental values are too large (see Supplemnetary Materials Table S3). For molar mass capability, no pronounced difference between the Zr and Hf species is predicted. Furthermore, the models indicate a slightly higher stereoselectivity and a lower regioselectivity for the Hf catalysts, based on a somewhat higher electrophilicity (regioselectivity) of the metal center and a slightly tighter active pocket (regioselectivity and stereoselectivity) due to ever-so-slightly shorter M–C distances; the exact opposite trend in selectivity is, however, observed experimentally (see Table 2**,** %*V*_Bur_ parameter). Considering that the QSAR models for *ansa*-zirconocenes rely predominantly (regioselectivity) or exclusively (molar mass capability, stereoselectivity) on steric factors, their failure to account for the Zr/Hf difference indicates that steric factors are not the origin of the “hafnium effect”.

**Figure 2:**
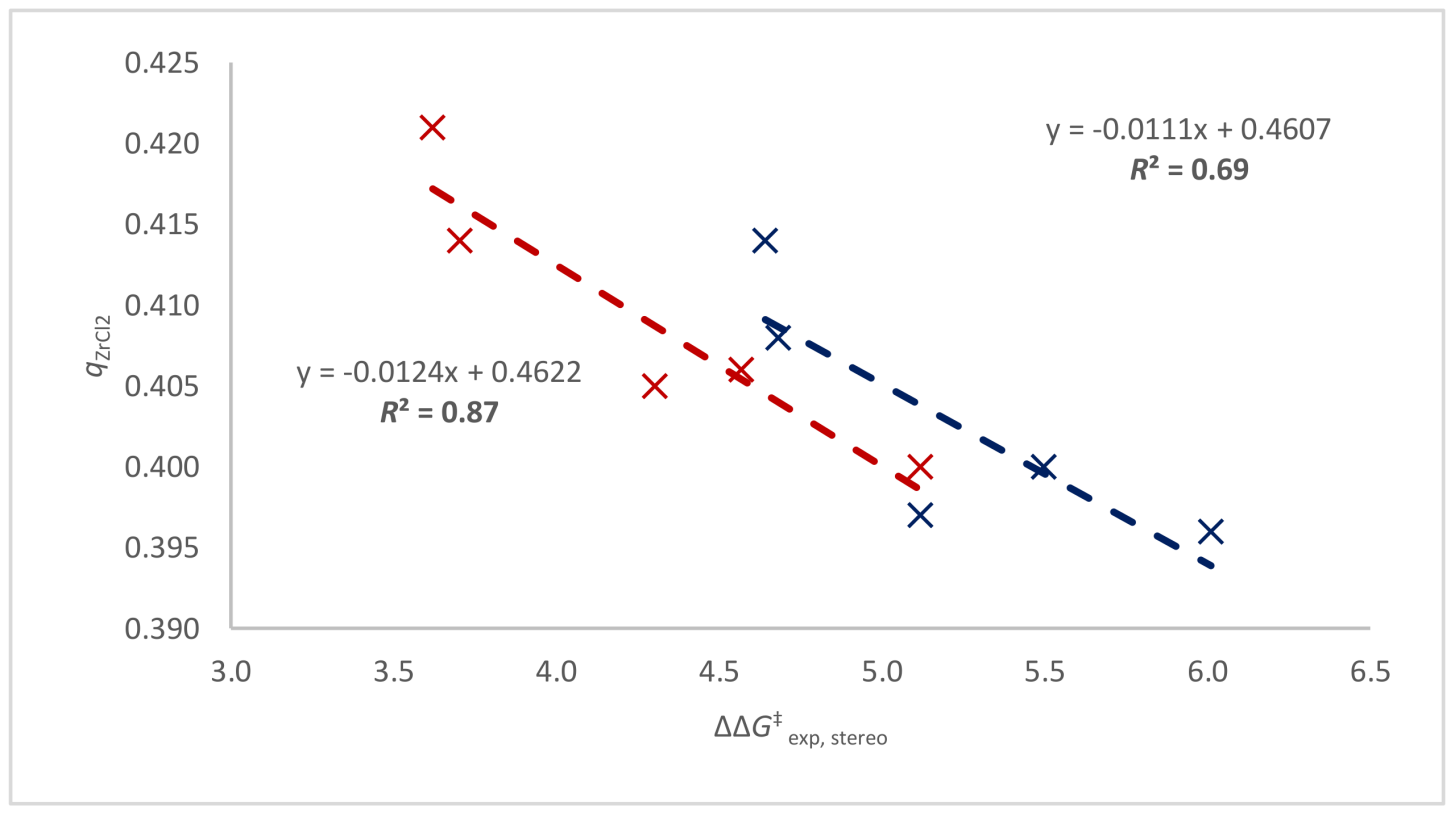
Correlation of experimentally observed stereoselectivity (ΔΔ*G*^‡^_exp, stereo_) at 100 °C and electrophilicity of catalyst dichloride precursor (as measured by *q*_MCl2_). Red trace hafnocenes **Hf-1** to **Hf-5**, blue trace zirconocenes **Zr-1** to **Zr-5**.

It should be noted that the stereoselectivity (ΔΔ*G*^‡^_exp, stereo_) of the hafnocenes at 100 °C follows solely electrophilicity trends, as measured by *q*_MCl2, NPA_ (*R*^2^ = 0.87; Figure 2), much better than zirconocenes (*R*^2^ = 0.69; Figure 2). As a matter of fact, the QSAR model for predicting the stereoselectivity of zirconocenes at 100 °C is based on the mathematical combination of two descriptors: (1) a 3D-steric descriptor, measuring the difference in buried volume of the occupied and empty quadrants of the catalyst active pocket (Δ%*V*_Bur,_ see Table 2) and (2) an electronic descriptor being the natural population analysis (NPA) charge on the metal dichloride fragment (*q*_ZrCl2, NPA_, see Table 1), introduced for accounting for the occurrence of chain epimerization [32]. The nice correlation for hafnocenes between ΔΔ*G*^‡^_exp, stereo_ and *q*_MCl2, NPA_ in Figure 2 indicates that at 100 °C chain epimerization is the dominant stereoerror source for Hf-based metallocenes.

Therefore, it appears that the different stereoselectivity of hafnocenes and zirconocenes simply reflects the slightly different electrophilicity of the two metals, with the more electrophilic Hf showing an earlier onset of chain epimerization via a series of BHE and reinsertion events. It should be noted here that variation of the substituents on the metallocene backbone influences the electrophilicity of the active species [32], but often in non-trivial ways, as direct electronic effects can be coupled with indirect electronic effects (e.g., from distortion).

#### 3.2.2. Modeling Key Differences between Hf/Zr

While absolute predictions for Hf catalysts using models for stereoselectivity, regioselectivity, and molar mass capability relying on Zr data (see also Table S3 and S4) are hampered by the inability to account correctly for the “hafnium effect”, two simple QSAR models can be built, capturing other key differences between Hf and Zr.

As previously discussed, the chain end analysis reported in Table 2 demonstrates that hafnocenes show a much higher tendency to undergo chain transfer via BME rather than BHE_T_. At *T*_p_ = 100 °C, 17% to 93% allyl chain ends are found in the polymers produced with the pairs **M-1** to **M-5**, with the remainder being vinylidene chain ends. This translates to a kinetic ratio of BME/BHE_T_ from 0.2 to 14 and, therefore, to differences in terms of activation of Gibbs free energy (ΔΔ*G*^‡^_BME-BHE__T, 373K_) between the two processes of −1.2 to 2.0 kcal/mol.

**Figure 3:**
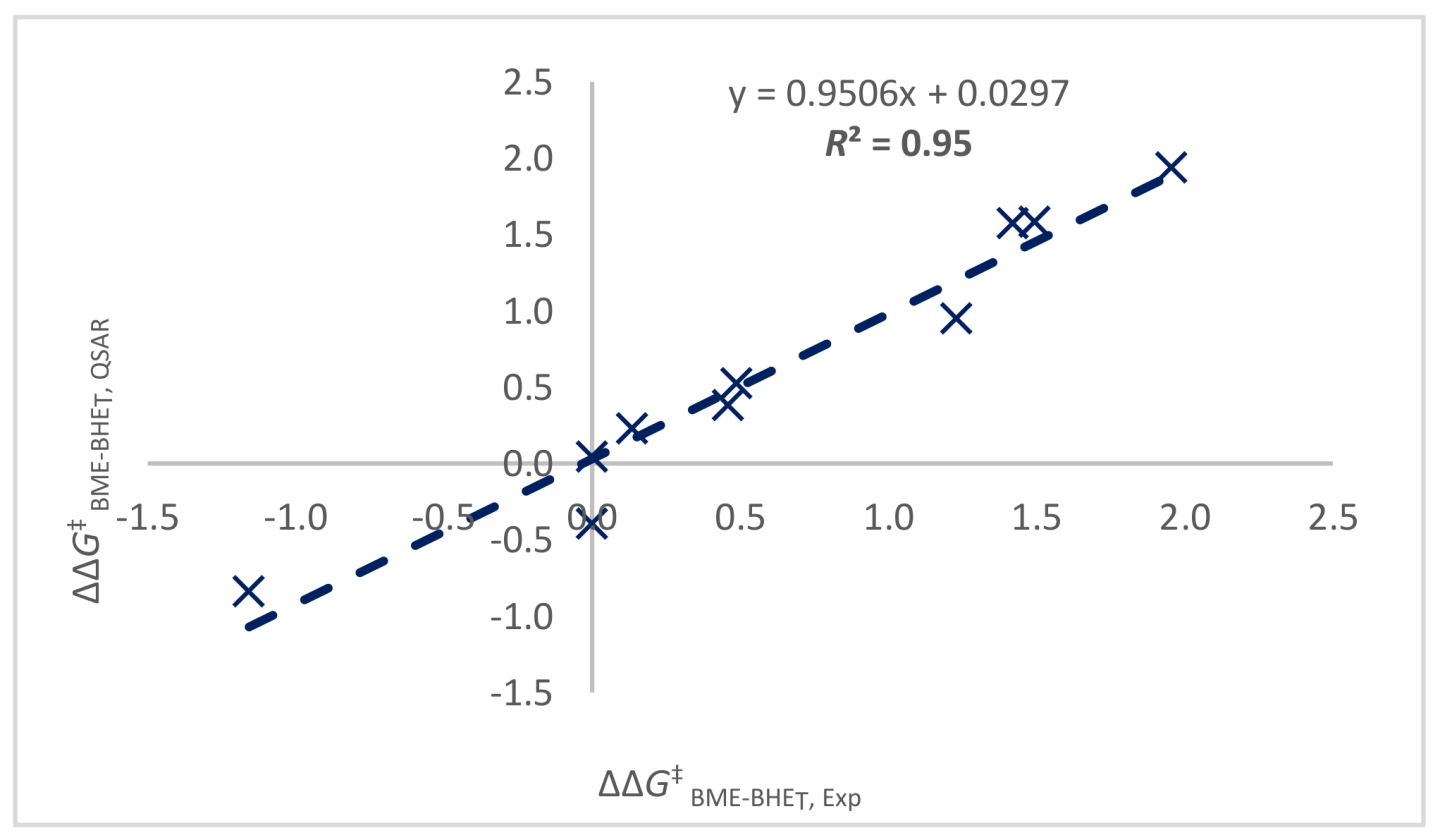
Correlation of experimentally observed ratio of allyl to vinyl chain ends (modeled as ΔΔ*G*^‡^_BME-BHET, Exp_) at 100 °C and QSAR-predicted ratio (ΔΔ*G*^‡^_BME-BHET, QSAR_) for catalysts **Hf-1** to **Hf-5** and **Zr-1** to **Zr-5**.

The penchant to prefer BME over BHE_T_ can be effectively modeled via a simple two-descriptor QSAR model for the whole dataset, i.e., for Hf and Zr alike. Along with the 3D steric descriptor Δ%*V*_Bur_, the best results were obtained here using the Hirschfeld charge on the MCl_2_ fragment (*q*_MCl2, Hirschfeld_) as the electronic descriptor for the whole catalyst set (*R*^2^ = 0.95, Figure 3 and Figure S5 and Table S5). A similar model can be built using the NPA charge *q*_MCl2, NPA_, resulting in a slightly worse but still satisfying agreement between experiments and calculations (*R*^2^ = 0.71, Figure S6). It is important to note that the electronic descriptor is dominant (*R*^2^ = 0.77 for *q*_MCl2, Hirschfeld_ alone, Figure S7), indicating that the electrophilicity of the metal center is the most important parameter determining the difference between Hf and Zr, also with respect to the preference for BME over BHE_T_.

**Figure 4:**
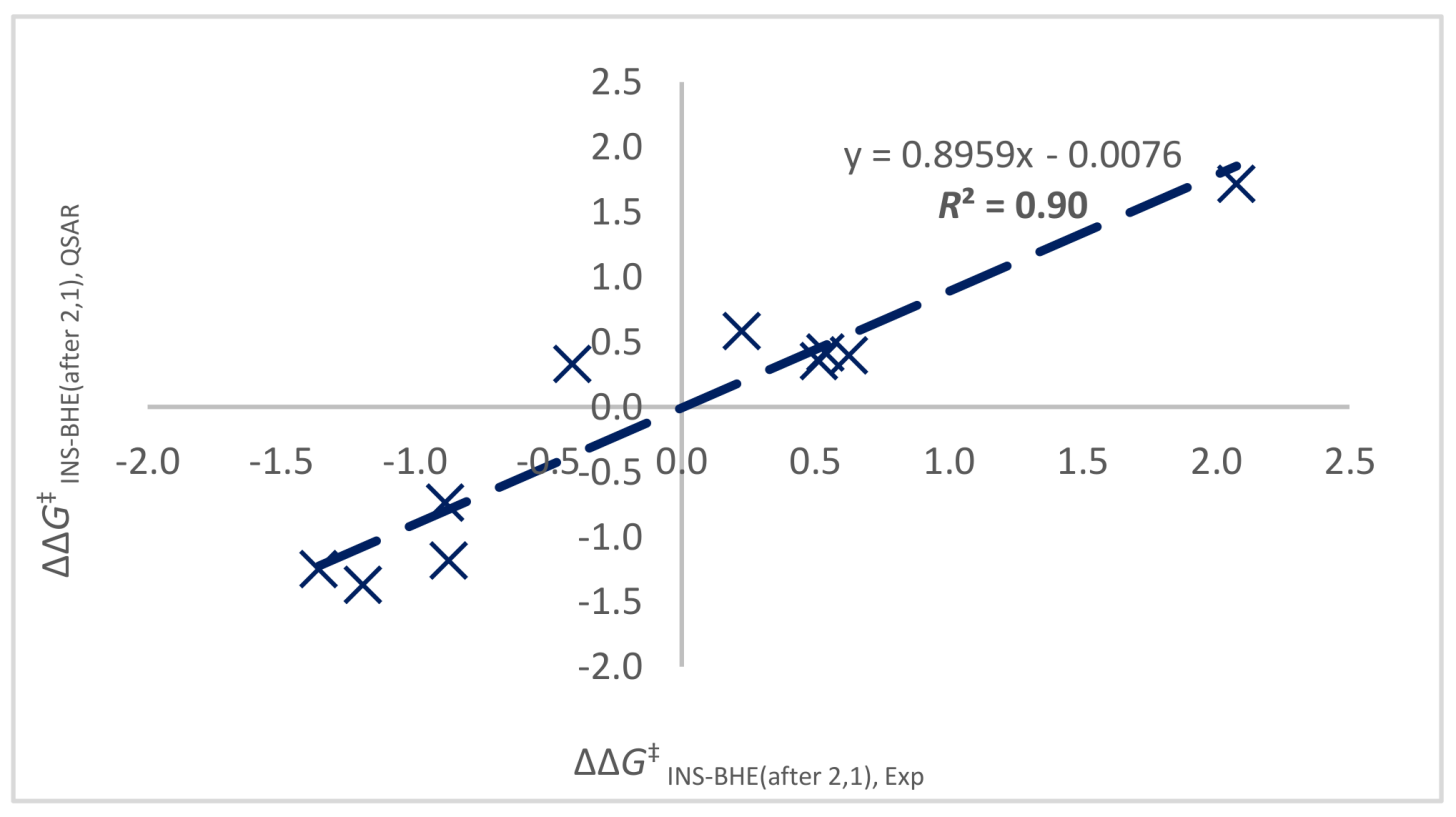
Correlation of experimentally observed ratio of 2,1 to 1,3 insertions (modeled as ΔΔ*G*^‡^_2,1INS-BHE, Exp_) at 100 °C and QSAR-predicted amount (ΔΔ*G*^‡^_2,1INS-BHE, QSAR_) for catalysts **Hf-1** to **Hf-5** and **Zr-1** to **Zr-5**.

The higher propensity of hafnocenes to isomerize 2,1 units into 3,1 units has the same origin. At *T*_p_ = 100 °C, a ratio of 2,1 to 3,1 regioerrors from 17 to 0.2 is found for the 10 metallocenes, indicating energy differences between 1,2 propagation after 2,1 insertion and 2,1 to 3,1 isomerization (ΔΔ*G*^‡^_1,2-2,1INS-BHE, 373K_) of −1.4 to 2.1 kcal/mol. A simple QSAR model based on the Δ%*V*_Bur_ descriptor and *q*_MCl2, Hirschfeld_ can be used to predict this ratio (*R*^2^ = 0.90, Figure 4 and Figure S8 and Table S6) for the combined Hf/Zr data set. Yet, again, the electronic descriptor is dominant (single-descriptor correlation *R*^2^ = 0.56).

The QSAR analysis, therefore, indicates that several simple catalyst performance indicators (stereoselectivity, prevalent chain transfer mechanism, tendency to isomerize regioerrors) have predominantly electronic origins. As Hf performs consistently worse at higher temperatures with respect to these performance indicators, we hypothesized that the stark performance decline of Hf-based metallocene has a common origin.

#### 3.2.3. Rationalizing the “Hafnium Effect”

As mentioned in the Introduction, recently, Rieger and coworkers proposed that the typical peculiarities applying to zirconium and hafnium in the coordinative polymerization of olefins originate from the different M–C bond characteristics and that the “hafnium effect” can be traced back to a higher enthalpic contribution to the activation barrier(s) in the case of Hf–C bond conversions [15]. Herein, we reanalyze this hypothesis in light of the performance of the five pairs of zirconocene and hafnocene catalysts studied and of the role that electronic factors play according to QSAR analysis, with particular emphasis on why the performance of Hf catalysts deteriorates faster than those of their Zr analogues at higher polymerization temperatures.

At the limit of very-low-polymerization temperatures and infinite propene concentration, one can confidently assert that molar mass capability of polymerization catalysts is fully determined by bimolecular processes. This means that chain transfer occurs via β-H transfer to the monomer (BHTM), which goes through a central transition state (TS) dominated by the making and breaking of M–C bonds [69]. Hf–C bonds have been reported to be somewhat stronger than Zr–C ones (e.g., by 5 kcal/mol in Cp *_2_MMe_2_) [70], resulting in higher barriers for BHTM in the former case. The longer polymers observed with Hf at low temperatures are, therefore, in line with Rieger’s M–C bond strength arguments [15].

Instead, at higher temperatures and/or moderate monomer pressures, as were applied in this study, monomolecular processes like BHE_T_ and/or BME typically become rate limiting for chain transfer. It is important to realize that BHE and BME are the microscopic reverse of insertion into an M–H or M–C bond, respectively. It is long known that olefin insertion TSs are geometrically very early for metallocenes; consequently, the elimination processes (the reverse) are late, and the corresponding TSs have a M–R(olefin) character (R = H or Me) [54,71]. This implies that M–C bonds’ strength arguments do not apply under these conditions as the M–C_chain_ bond is barely elongated and the M-C_olefin_ bond barely formed (or, viewed from the elimination point of view, the bonds are fully formed and completely broken, respectively).

The Hammond postulate [72] provides the grounds to understand the faster performance deterioration with Hf. Given the late geometric nature of the TSs, stabilizing the M–R(olefin) product over the M–R starting species will lower the barrier for BHE to a similar degree because highly electrophilic early transition metal complexes are stabilized in the former case by two electron-donating ligands (R and olefin) as opposed to only one (R). The relative stabilization increases the more electrophilic the active species is. This explains the higher propensity of the more electrophilic Hf species to undergo chain epimerization and to isomerize 2,1 into 3,1 units, since BHE events are the rate-limiting steps for both processes (Figure 3 and Figure 4).

To justify the more pronounced molecular weight decline of hafnocenes with respect to zirconocenes, it is important to separate the relative propensity for BHE from BHE_T_ events and compare the latter with BME events. The combined experimental data of Table 1 and Table 2 show that, for both Hf and Zr, BHE events are more likely than BME. Specifically, from the ratio between BME-derived allyl chain ends and the sum of all microstructural fragments formed via BHE after 1,2 insertion (i.e., vinyl chain ends, internal olefins, and stereoerrors from chain epimerization; Scheme 1), it can be estimated that for zirconocenes approximately five, and for hafnocenes eight BHE events occur every 1 BME at 100 °C. This is calculated assuming that all stereoerrors stem from chain epimerization, which is reasonable for Hf but not fully backed up by the data and models for Zr. However, the ratio changes only lightly to 4 if stereoerrors are not counted for Zr.

Despite the fact the Hf species are more prone to BHE, as previously discussed, only 3% of the overall BHE events led to chain transfer (BHE_T_), vs. 15% in the case of Zr species. Consequently, hafnocenes showed a higher tendency than zirconocenes to undergo chain transfer via BME rather than BHE_T._

Overall, this analysis indicates that hafnocenes have a higher preference for BHE than zirconocenes, but a lower tendency to release the chain from the resulting M–H(vinyl-polymer) species (BHE_T_). This difference can be ascribed to the higher M–H(olefin) resting-state stabilization for Hf than for Zr, i.e., stronger olefin coordination. For hafnocenes at high temperature, BME is the main chain transfer mechanism, not BHE_T_. However, this is not a result of selective lowering of the BME barrier but because BHE events rarely lead to chain release for Hf. In fact, both BHE (general) and BME barriers are lower for Hf in comparison to Zr as the result of an electrophilicity effect. These rationalizations are gratifyingly in line with the combined experimental/QSAR analysis discussed in the previous sections.

Reproducing experimental evidence quantitatively by computational methods is challenging since the required accuracy is beyond the limits of static DFT methodologies (chemical accuracy, ±1 kcal/mol) and one should note that, for example, Laine et al. concluded the opposite from non-dispersion-corrected B3LYP calculations [73].

**Figure 5:**
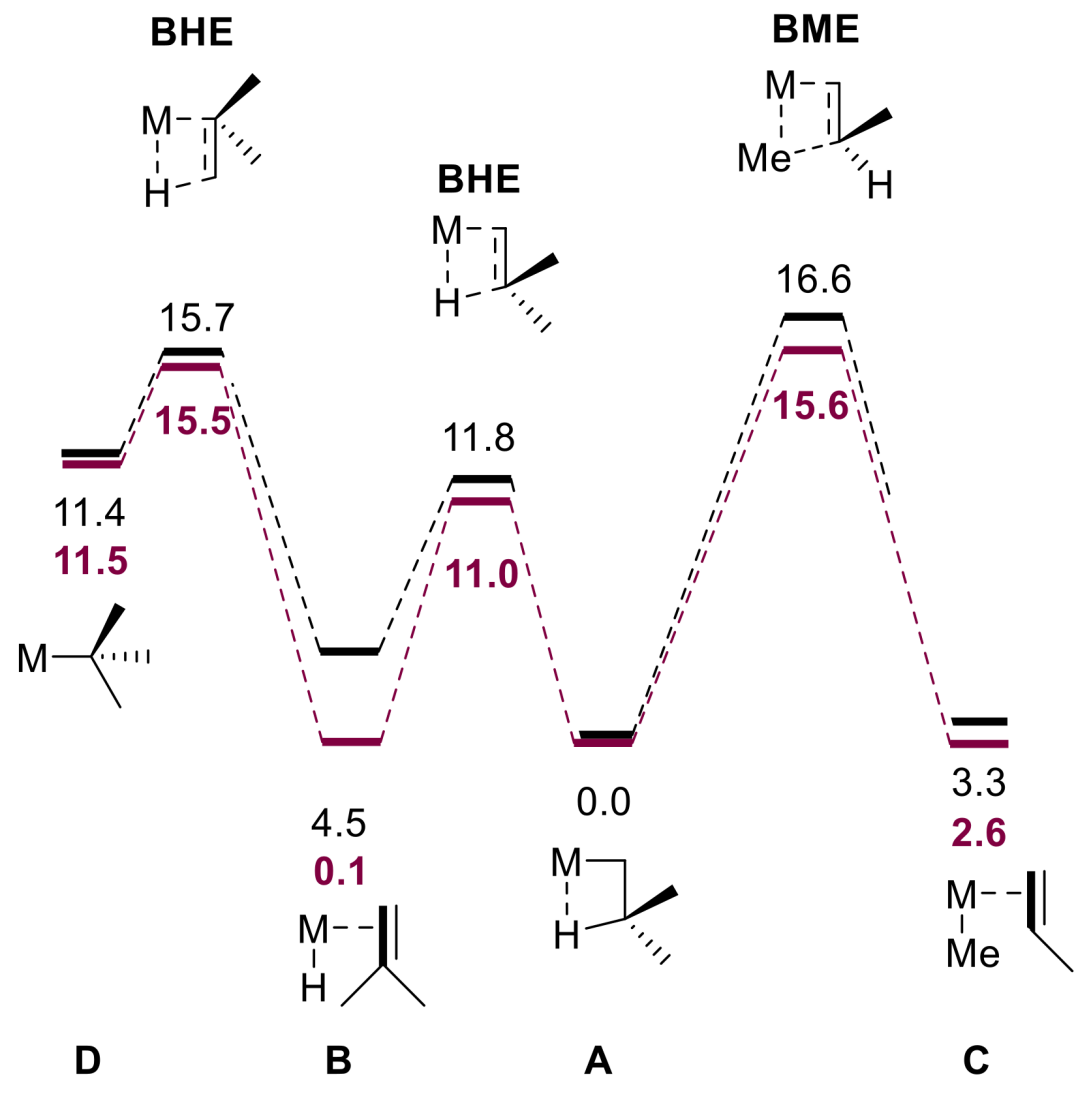
Potential energy surface for BHE and BME events in the **M-2** catalyst pair (ligands omitted). Level of theory MN15(SMD)/cc-pVTZ-(PP)//MN15/cc-pVDZ-(PP), *T* = 100 °C, Gibbs free energies in kcal/mol. Black traces and numbers for **Zr-2**, burgundy traces and numbers for **Hf-2**.

However, employing a “modern” dispersion-aware functional (MN15) to compare BME and BHE barriers in **Zr-2** with **Hf-2** produces the energy profiles shown in Figure 5 (see also Tables S7 and S8). The results show that the olefin-coordinated intermediates are stabilized by 1–4 kcal/mol for **Hf-2** with respect to **Zr-2**. The associated BHE and BME barriers are lowered in the Hf case, in accordance with the Hammond postulate and the proposed origin of the higher temperature “hafnium effect”.

## 4. Conclusions

The different performance of hafnium- and zirconium-based olefin polymerization catalysts has long puzzled chemists. The set of five Hf/Zr *C_2_*-symmetric metallocene pairs screened in this work, including some of the best high-temperature polymerization catalysts known today, provides a rather robust comparison of Hf vs. Zr but also of different ancillary ligands at medium- to high-polymerization temperature.

Chemically meaningful QSAR models developed from the polymerization data garnered at *T*_p_ =100 °C for stereoselectivity, the ratio of allyl to vinyl terminations, and the ratio of 2,1 to 3,1 insertions in the polymer indicate a strong dependence of catalyst performance on electrophilicity, which is a function of both the central metal and the ligand framework. A rationale can be found for this observation based on a Hammond analysis of transition states: More electrophilic active species lead to more stable olefin complexes, resulting in lower barriers for BHE and BME and higher barriers for vinyl- or allyl-terminated chain release thereafter. These two factors cooperate in determining the ease of chain termination and rearrangements (i.e., chain epimerization and 2,1- to 3,1-unit isomerization).

Based on this insight, the stronger performance decline of hafnocenes can be traced to a stabilization effect of olefin-coordinated resting states and their associated transition states. At high temperatures, the ease of all elimination processes increases more rapidly for the more electrophilic hafnocenes, and it is only partially compensated by the higher barriers for chain release. Consequently, only the most electron-donating ligand frameworks, like those of **Hf-3**, **Hf-4,** and **Hf-5**, can mitigate this effect and yield hafnocenes that outperform their corresponding zirconocene counterparts, also at industrially relevant high-polymerization temperatures.

## Figures and Tables

**Table 1:** Results of the characterization of iPP samples prepared at *T*_p_ = 60 °C and *p*(C_3_H_6_) = 6.6 bar or *T*_p_ = 100 °C and *p*(C_3_H_6_) = 7.9 bar with the five catalyst pairs of Figure 1 (**M-1** to **M-5**; see text for details).

	60 °C							100 °C						
ID	2,1 %	3,1 %	*regio*_tot_^#^ %	1-σ ^≠^ %	*M*_n_ KDa	*M_w_/M_n_*	*T*_m_ °C	2,1 %	3,1 %	*regio*_tot_^#^ %	1-σ ^≠^ %	*M*_n_ KDa	*M_w_/M_n_*	*T*_m_ °C
**Zr-1 ^†^**	0.17	n.d.	0.17	0.06	530	2.2	162.4	0.21	0.09	0.30	0.18	60	2.0	153.9
**Hf-1**	0.06	0.02	0.08	0.14	390	2.1	162.6	0.02	0.10	0.12	0.67	31	2.0	151.9
**Zr-2** ^†^	0.32	0.09	0.42	0.02	470	1.8	158.2	0.19	0.33	0.52	0.19	48	1.9	152.2
**Hf-2**	0.15	0.09	0.24	0.10	210	2.4	158.8	0.04	0.25	0.28	0.75(6)	16	2.0	149.1
**Zr-3** ^†^	0.23	n.d.	0.23	0.03	400	2.2	162.2	0.29	0.14	0.43	0.10	44	2.0	154.6
**Hf-3**	0.07	n.d.	0.07	0.17	1500	2.2	165.5	0.04	0.13	0.17	0.30	60	2.0	156.0
**Zr-4** ^†^	0.67	0.04	0.71	0.02	1400	2.3	156.0	0.57	0.42	0.99	0.06	160	2.2	149.5
**Hf-4**	0.38(3)	0.12	0.50(4)	0.07	1900	2.2	156.1	0.13	0.42	0.56(3)	0.21	140	2.0	151.6
**Zr-5** ^†^	0.26	n.d.	0.26	≤0.02	(1100) ^=^	2.5	163.2	0.33	0.02	0.35	0.03	200	2.1	157.4
**Hf-5**	0.11	n.d.	0.11	0.03	1400	2.3	164.2	0.12	0.06	0.18	0.10	90	2.0	158.7

Experimental uncertainty is ±2 on the last significant digit for 1-*σ* and *regio*_tot_, unless otherwise indicated in parentheses, and ±20% on *M*_n_. For more details, see Supplementary Materials Tables S1 and S2. ^†^ 60 and 100 °C data are taken from [23,24,25,32]. ^#^ Total fraction of 2,1 and 3,1 monomeric units in % (as determined by ^13^C NMR). ^≠^ Fraction of stereoirregular monomeric units in %, according to the enantiomorphic site statistical model [63]. ^=^ **Zr-5** is exceedingly hard to control at low-polymerization temperature and the *M*_n_ is likely underestimated due to diffusion limitations [24] (see text for details).

**Table 2:** NMR chain end analyses (as fraction of all monomeric units in %) of the polymers produced at *T*_p_ = 100 °C and *p*(C_3_H_6_) = 7.9 bar with the five catalyst pairs (**M-1** to **M-5**) of Figure 1.

ID	Δ%*V*_Bur_ *	*q* ^−^_MCl2_^†^	Vinylidene, %	Internal Olefin, %	Allyl, %	*x*_Allyl_^#^
**Zr-1**	16.8	0.408	0.220	0.113	0.220	0.50
**Hf-1**	16.9	0.414	0.020	0.030	0.105	0.84
**Zr-2**	17.6	0.414	0.050	0.072	0.060	0.55
**Hf-2**	17.9	0.421	0.032	0.042	0.240	0.88
**Zr-3**	16.2	0.397	0.062	0.043	0.013	0.17
**Hf-3**	16.5	0.405	0.042	0.025	0.081	0.66
**Zr-4**	17.6	0.400	0.007	0.022	0.007	0.50
**Hf-4**	18.4	0.406	0.005	0.014	0.034	0.87
**Zr-5**	19.2	0.396	0.007	0.027	0.013	0.65
**Hf-5**	20.0	0.400	0.003	0.032	0.042	0.93

* Buried volume parameter Δ%*V*_Bur_ measuring the difference between the occupied and the empty quadrants of the catalyst using a 5.0 Å scanning sphere centered on the central metal. ^†^ Natural population analysis (NPA) charge on the metal dichloride fragment determined by DFT. ^#^ Molar fraction of allyl-type chain ends.

## Data Availability

Not applicable.

## Supplementary Materials

The following are available online at https://www.mdpi.com/article/10.3390/polym13162621/s1, Figure S1, Exemplary Rapid-GPC curves for i-PP samples produced with catalysts Hf-1 to Hf-2 at 60 °C, Figure S2, Exemplary Rapid-GPC curves for i-PP samples produced with catalysts Hf-1 to Hf-2 at 100 °C, Figure S3, ^13^C NMR spectra of some representative samples obtained with catalysts Hf-1 to Hf-5 at 60 °C, Figure S4, ^13^C NMR spectra of some representative samples obtained with catalysts Hf-1 to Hf-5 at 100 °C, Figure S5, Analysis of variance, chain end termination model, Figure S6, Correlation of experimentally observed ratio of allyl to vinyl chain ends at 100 °C and QSAR predicted ratio for catalysts Hf-1 to Hf-5 and Zr-1 to Zr-5, using NPA rather than Hirschfeld charges, Figure S7, Single descriptor correlation of q_ZrCl2,HF_ with ΔG^‡^_BME-BHET_, Figure S8, Analysis of variance, 1,2-to-1,3 regioerror isomerization Model, Table S1, Stir paddles, scavenger amounts, propene pressure and solvent choice for the polymerization experiments, Table S2, Propene Polymerization Experiments (PPR), Table S3, Predicted vs. observed performance in of Hf-1 to Hf-5 at 60 °C, Table S4, Predicted vs. observed performance Hf-1 to Hf-5 at 100 °C, Table S5, Computational descriptors, experimental performance indicator, and experimental and QSAR predicted ΔG^‡^_BME-BHET_, Table S6, Computational descriptors, experimental performance indicator, and experimental and QSAR predicted ΔΔG^‡^_1,2-2,1INS-BHE_, Table S7, Final energies, entropy and enthalpy corrections, Table S8, Relative enthalpies and Gibbs free energies, Combined XYZ file, LMCL_2_ and TS structures, experimental procedures for synthesis of Hf-1 to Hf-5 and detailed polymerization procedures.
