# Supplementary material for: Hafnium vs. Zirconium, the Perpetual Battle for Supremacy in Catalytic Olefin Polymerization: A Simple Matter of Electrophilicity?
Source: Polymers (Basel). 2021 Aug 6;13(16):2621. doi: 10.3390/polym13162621 (PMC8400551; doi:10.3390/polym13162621)
Supplement: Supplementary file 1 [file polymers-13-02621-s001.zip › polymers-1316873-supplementary.pdf]

# SUPPORTING INFORMATION

## *Hafnium vs Zirconium, The Perpetual Battle for Supremacy in Catalytic Olefin Polymerization: A Simple Matter of Electrophilicity?*

*Antonio Vittoria,[1] Georgy P. Goryunov,[2] Vyatcheslav V. Izmer,[2] Dmitry S. Kononovich,[2] Oleg V. Samsonov,[2] Francesco Zaccaria,[3] Gaia Urciuoli,[1] Peter H. M. Budzelaar,[1] Vincenzo Busico,[1] Alexander Z. Voskoboynikov,[2] Dmitry V. Uborsky,[2*] Christian Ehm[1*] and Roberta Cipullo[1*]*

[1] Dipartimento di Scienze Chimiche, Università di Napoli Federico II, Via Cintia, 80126 Napoli, Italy;

[2] Department of Chemistry, Lomonosov Moscow State University, 1/3 Leninskie Gory, 119991 Moscow, Russia;

[3] Dipartimento di Chimica, Biologia e Biotecnologie and CIRCC, Università di Perugia, 06123 Perugia, Italy

[*] Correspondence: duborsky@med.chem.msu.ru (D.V.U.); christian.ehm@unina.it (C.E.); rcipullo@unina.it (R.C.)

## Contents

1. Synthesis of complexes ........ 2
   1.1 General Details ........ 2
2. Polymerization Experiments and Polymer Characterization ........ 6
   2.1 Experimental procedures in the PPR platform ........ 6
   2.2 Polymer workup (PPR) ........ 7
   2.3 Polymer analytical characterizations ........ 7
3. QSAR Modeling ........ 9
   3.1 QSAR Modeling – Equations for Stereo-, Regioselectivity and Molecular Weight Capability Models ........ 11
   3.2 Predicted vs. observed performance at $T_p$ = 100°C for **Hf-1 to Hf-5** ........ 12
   3.3 QSAR Models for Chain End Termination and Propensity for 1,2-to-1,3 regioerror Isomerization ........ 13
   3.4 Final Energies, Enthalpies and Free Energies for species in Figure 5. ........ 16
4. References ........ 17

# 1. Synthesis of complexes

## 1.1 General Details

All manipulations with compounds, which are sensitive to moisture and air, were performed either in an atmosphere of argon using a standard Schlenk technique or in an inert atmosphere (Ar) of glove box (MBraun). $^{1}H$, $^{13}C$ and $^{19}F$ spectra were recorded on Bruker Avance-400 spectrometer for 1–10% solutions in deuterated solvents. Chemical shifts for $^{1}H$ and $^{13}C$ are reported relatively to TMS and referenced to the residual $^{1}H$ or $^{13}C$ resonances of the deuterated solvents. C, H microanalyses were done using a «Elementar Vario MICRO cube» analyzer.

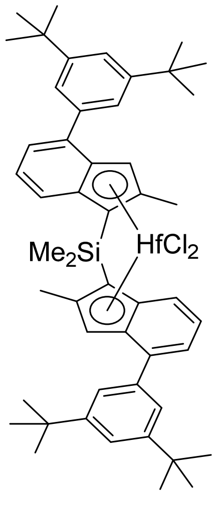


**Synthesis of Hf-1.** To a solution of 5.20 g (7.5 mmol) of bis[4-(3,5-di-*tert*-butylphenyl)-2-methyl-1*H*-inden-1-yl](dimethyl)silane in 250 mL of ether, 6.0 mL *n*BuLi (2.5 M in hexane, 15.0 mmol) was added at 0 °C. The mixture was stirred for 12 h at room temperature, cooled to -80 °C, and 2.40 g of $HfCl_4$ (7.5 mmol) was added. After that, the ether was evaporated, and the residue was taken up in hot toluene (100 mL), and filtered (G4), the filter cake was washed with additional 2×20 mL of hot toluene. The filtrate was evaporated to ~50 mL volume, and 30 mL of n-hexane was added. The precipitated solid was filtered and dried in vacuum. This gave 0.19 g (3%) of **Hf-1**. Anal. Calcd for $C_{50}H_{62}Cl_2HfSi$: C, 63.85; H, 6.64. Found: C, 64.03; H, 6.75. $^{1}H$ NMR (400 MHz $C_6D_6$) $\delta$ 7.70 (d, $J$ = 8.7 Hz, 2H), 7.53 (d, $J$ = 1.7 Hz, 4H), 7.41 (m, 2H), 7.40 (d, $J$ = 7.0 Hz, 4H), 7.09 (dd, $J$ = 8.7, 7.0 Hz, 2H), 6.90 (s, 2 H), 2.36 (s, 6H), 1.34 (s, 6H), 1.32 (s, 36H). $^{13}C\{^{1}H\}$ NMR (100 MHz, $CDCl_3$): $\delta$ 150.6, 139.0, 138.2, 132.6, 131.4, 125.8, 125.7, 125.2, 123.6, 123.0, 121.1, 120.8, 83.9, 34.7, 31.1, 18.1, 2.2.

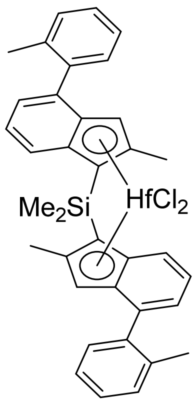


**Synthesis of Hf-2**. To a solution of 6.34 g (12.8 mmol) of bis[4-(2-methylphenyl)-2-methyl-1*H*-inden-1-yl](dimethyl)silane in 250 mL of ether, 10.2 mL *n*BuLi (2.5 M in hexane, 25.5 mmol) was added at 0 °C. The mixture was stirred for 2 h at room temperature, cooled to -80 °C, and 4.09 g $HfCl_4$ (12.8 mmol) was added. The mixture was then stirred overnight at room temperature. The obtained suspension was filtered while hot through a pad of Celite; the filter cake was washed with additional 2×20 mL of hot toluene. The filtrate was evaporated to ~40 mL volume, and 40 mL of n-hexane was added. The precipitated solid was filtered and dried in vacuum. This gave 0.64 g (6%) of **Hf-2** containing 0.5 equivs of cocrystallized toluene. Anal. Calcd for $C_{36}H_{34}Cl_2HfSi\cdot 0.5C_7H_8$: C, 60.04; H, 4.85. Found: C, 59.41; H, 4.72. $^{1}H$ NMR (400 MHz, $CDCl_3$) $\delta$ 7.68 (d, $J$ = 8.7 Hz, 2H), 7.53 (br. s., 2H), 7.28–7.21 (br. s., 6H), 7.14–7.20 (m, 2H), 7.05 (dd, $J$ = 8.7, 6.9 Hz, 2H), 6.43 (br. s., 2H), 2.33 (m, 6H), 2.06 (br. s., 6H), 1.32 (s, 6H). $^{13}C\{^{1}H\}$ NMR (101 MHz, $C_6D_6$): $\delta$ 139.9, 138.0, 135.9, 134.4, 133.1, 130.6, 130.5, 129.7, 128.9, 126.9, 125.9, 125.8, 124.7, 121.2, 84.9, 20.5, 18.8, 2.5.

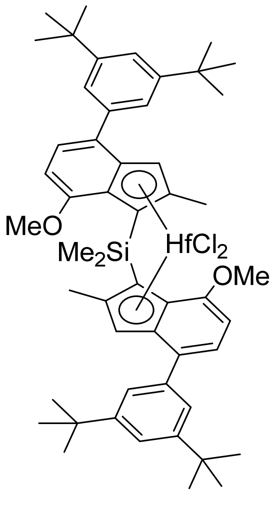

**Synthesis of Hf-3.** To a solution of 10.21 g (13.56 mmol) of bis[4-(3,5-di-*tert*-butylphenyl)-7-methoxy-2-methyl-1*H*-inden-1-yl](dimethyl)silane in 200 mL of ether, 11.2 mL (27.22 mmol) of 2.43 M *n*BuLi in hexanes was added in one portion at -50 °. The mixture was stirred for 4 h at room temperature, then cooled to -50 °C and 4.35 g (13.58 mmol) of $HfCl_4$ was added. The mixture was stirred for 48 h at room temperature and then evaporated almost to dryness. The residue was taken up in 350 mL of hot toluene. The obtained mixture was filtered while hot through glass frit (G4) and the filtrate was evaporated to ~250 mL volume. A yellow crystalline solid precipitated from this solution after standing overnight at room temperature was collected and dried in vacuum. This gave 7.19 g of *meso*-**Hf-3** containing cocrystallized toluene, which was equal to 6.51 g (48%) of pure *meso*-**Hf-3**. The mother liquor was evaporated to ~50 mL volume. A yellow crystalline solid precipitated from this solution after standing overnight at room temperature was collected and dried in vacuum. This gave an additional 1.3 g of *meso*-**Hf-3** containing cocrystallized toluene, which was equal to 1.18 g (9%) of pure *meso*-**Hf-3**. The mother liquor was evaporated to a highly viscous oil, which was triturated with 40 mL of n-hexane. Yellow precipitate formed was filtered off and dried in vacuum. This procedure gave 1.40 g (10%) of a ~1/1 mixture of **Hf-3** (racemate) and *meso*- **Hf-3**. The total yield of **Hf-3** and *meso*-**Hf-3** was 67%. *meso*-**Hf-3**, $^1$H NMR (400 MHz, $CDCl_3$): $\delta$ 7.46 (d, $J$ = 1.7 Hz, 4H), 7.37 (t, $J$ = 1.7 Hz, 2H), 7.10 (d, $J$ = 7.8 Hz, 2H), 6.82 (s, 2H), 6.20 (d, $J$ = 7.8 Hz, 2H), 3.88 (s, 6H), 2.61 (s, 6H), 1.34 (s, 36H), 1.32 (s, 3H), 1.25 (s, 3H).

*Isomerization of meso-***Hf-3** *to* **Hf-3.** To a mixture of 7.19 g (6.51 g excluding cocrystallized toluene, 6.51 mmol) of *meso*-**Hf-3** and 600 mg (14.2 mmol) of LiCl 150 mL of THF was added. This mixture was stirred for 48 h at 64 °C. That resulted in almost quantitative isomerization of *meso*-**Hf-3** to racemic **Hf-3**. The mixture was evaporated to dryness, the solid residue was taken up in 400 mL of hot toluene. The obtained suspension was filtered while hot through glass frit (G4) and the filtrate was evaporated to ~50 mL volume. A yellow crystalline solid precipitated from this solution after standing overnight at room temperature was collected and dried in vacuum. This gave 5.12 g of **Hf-3**. The mother liquor was evaporated almost to dryness and the residue was triturated with 40 mL of n-hexane. A yellow precipitate thus formed was filtered off and dried in vacuum. This gave an additional 1.07 g of **Hf-3**. Thus, the total yield of **Hf-3** from *meso*-**Hf-3** was 6.19 g (95%). The analytical data matched that from the literature.[1]

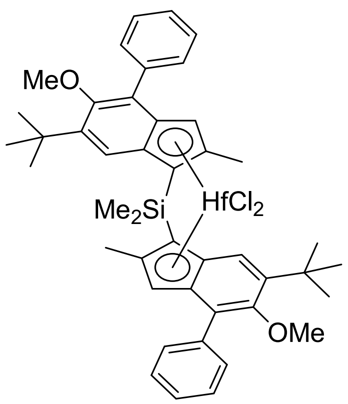

**Synthesis of Hf-4.** To a suspension of 11.6 g (18.1 mmol) of bis(6-*tert*-butyl-5-methoxy-2-methyl-4-phenyl-1*H*-inden-1-yl)(dimethyl)silane in 150 mL of $Et_2O$, 14.9 mL (36.2 mmol) of 2.43 M *n*BuLi in hexanes was added in one portion at -50 °C. The mixture was stirred overnight at room temperature, then cooled to -50 °C and 5.8 g (18.1 mmol) of $HfCl_4$ was added. The mixture was stirred for 24 h at room temperature and then evaporated to dryness. The residue was taken up in 150 mL of hot toluene and the mixture was filtered while hot through glass frit (G4). The filtrate was evaporated to ~50 mL volume. A yellow solid precipitated after standing overnight at room

temperature was filtered and dried in vacuum. This gave 3.3 g (21%) of **Hf-4**. The mother liquor was evaporated to ~30 mL volume. A yellow solid precipitated after standing overnight at room temperature was filtered and dried in vacuum. This gave an additional 6.4 g (40%) of **Hf-4**. The mother liquor was evaporated to an oil which was triturated with 20 mL of n-hexane. A precipitated yellow solid was filtered and dried in vacuum to give an additional 2.38 g (15%) of **Hf-4**. The total yield **Hf-4** was 12.08 g (75%). **Hf-4**: Anal. calc. for $C_{44}H_{50}Cl_2HfO_2Si$: C, 59.49; H, 5.67. Found: C, 59.84; H, 5.81. $^1$H NMR (400 MHz, $CDCl_3$): $\delta$ 7.61 (very br.s, 4H), 7.56 (s, 2H), 7.43 (dd, *J* = 7.4 Hz, 4H), 7.32 (t, *J* = 7.4 Hz, 2H), 6.50 (s, 2H), 3.39 (s, 6H), 2.25 (s, 6H), 1.39 (s, 18H), 1.28 (s, 6H). $^{13}C\{^1H\}$ NMR (101 MHz, $CDCl_3$): $\delta$ 159.8, 143.8, 137.0, 133.1, 132.5, 129.7, 128.5, 127.2, 126.5, 121.3, 121.1, 119.1, 82.9, 62.7, 35.7, 30.4, 18.2, 2.4.

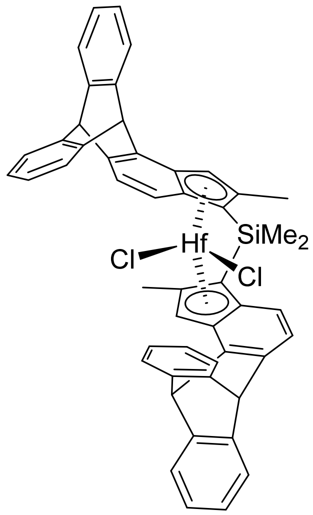


**Synthesis of Hf-5-Cl₂**. To a solution of 1.55 g (2.32 mmol) of bis(2-methyl-*1H*-cyclopenta[a]triptycene-1-yl)(dimethyl)silane in 50 ml of diethyl ether, 1.85 ml (4.63 mmol) of 2.5 M *n*BuLi in hexanes was added at room temperature. This mixture was stirred overnight, and then 1.08 g (2.32 mmol) of $HfCl_4(THF)_2$ was added in one portion at -80 °C. The resulting mixture was stirred for 24 h at room temperature and then evaporated to dryness. Further on, 50 ml of toluene was added. The resulting mixture was heated to 110 °C and filtered through the short pad of Celite 503. The filtrate was evaporated to dryness. The residue was recrystallized from toluene giving 610 mg (28%) of mixture of *rac*- **Hf-5-Cl₂** and *meso*- **Hf-5-Cl₂** (7:1 molar ratio) as yellow crystals. *Rac*-isomer: $^1$H NMR (400 MHz, $CDCl_3$): δ (ppm) 7.20-7.41 (m, 14H), 6.90-7.03 (m, 6H), 6.72 (s, 2H), 5.49 (s, 2H), 5.48 (s, 2H), 2.23 (s, 6H), 1.16 (s, 6H). $^{13}C\{^1H\}$ NMR (100 MHz, $CDCl_3$): $\delta$ 146.30, 146.2, 145.2, 143.6, 141.1, 133.5, 130.8, 129.0, 128.2, 125.3, 124.75, 124.73, 124.65, 124.5, 123.9, 123.36, 123.31, 122.7, 122.6, 121.7, 115.5, 84.3, 54.4, 51.4, 18.6, 2.5.

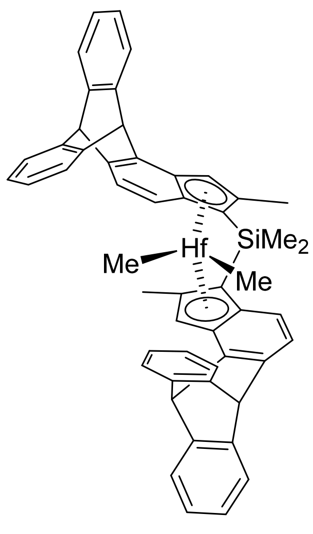


**Synthesis of Hf-5.** To a suspension of 500 mg (0.545 mmol) of the of *rac*-**Hf-5-Cl₂** and *meso*-**Hf-5-Cl₂** (7:1 molar ratio) in 40 ml of toluene 570 ul of 2.9 M MeMgBr (1.64 mmol) in ether was added at room temperature. This mixture was stirred for 24 h at 100 °C and then evaporated to dryness in vacuum. To the residue 40 ml of toluene were added and the mixture was heated up to 110 °C and then was passed while hot through a short pad of Celite 503. The filtrate was evaporated to dryness. According to $^1$H NMR analysis a pure mixture of dimethyl complexes **Hf-5** (racemate) and *meso*-**Hf-5** (molar ratio 7:1) was obtained. The residue was recrystallized from toluene/hexane mixture giving 190 mg (37%) of pure racemic **Hf-5** containing 0.5 equivs of cocrystallized toluene. $^1$H NMR (400 MHz, $CDCl_3$): δ (ppm) 7.31-7.35 (m, 6H), 7.27 (d, *J* = 7.0 Hz, 2H), 7.22 (d, *J* = 8.1 Hz, 2H), 7.10 (d, *J* = 8.6 Hz, 2H), 6.96 (dt, *J* = 1.16, 7.3 Hz, 2H), 6.87-6.92 (m, 6H), 6.69 (s, 2H), 5.52 (s, 2H), 5.43 (s, 2H), 2.04 (s, 6H), 0.96 (s, 6H), -2.23 (s, 6H). $^{13}C\{^1H\}$ NMR (100 MHz, $CDCl_3$): $\delta$ 146.8, 146.4, 145.5,

143.9, 143.3, 140.7, 133.8, 129.0, 128.2, 127.3, 125.33, 125.28, 124.78, 124.6, 124.2, 123.3, 122.8, 121.7, 120.9, 110.6, 80.9, 54.3, 51.7, 39.7, 18.0, 2.6.

# 2. Polymerization Experiments and Polymer Characterization

## 2.1 Experimental procedures in the PPR platform

Propene polymerization experiments were performed in a Freeslate Parallel Pressure Reactor setup with 48 reaction cells (PPR48), fully contained in a triple MBraun glovebox operating under nitrogen. The cells, each with a liquid working volume of 5.0 mL, featured an 800 rpm magnetically coupled stirring, and individual online reading/control of temperature, pressure, monomer uptake, and monomer uptake rate. Experiments were carried out according to established experimental protocols[2–4] detailed below.

Prior to the execution of a polymerization library, the PPR modules undergo 'bake-and-purge' cycles overnight (8 h at 90-140°C with intermittent dry $N_2$ flow), to remove any contaminants and left-overs from previous experiments. After cooling to glovebox temperature, the module stir tops are taken off, and the 48 cells are fitted with disposable 10 mL glass inserts (pre-weighed in a Mettler-Toledo Bohdan Balance Automator) and stir paddles (Table S1). The stir tops are then set back in place, and $N_2$ in the reactors is replaced with propene (ambient pressure). The cells are then loaded with the appropriate amounts of solvent containing TIBA as a scavenger (Table S1 and S2).

**Table S1.** Stir paddles, scavenger amounts, propene pressure and solvent choice for the polymerization experiments.

| **Condition** | ***T*$_p$ = 60°C** | ***T*$_p$ = 100°C** |
|---|---|---|
| **Stir Paddles** | polyether ether ketone (PEEK) | Titanium |
| **TiBA Scavenger** | 10 μmol | 10 μmol |
| **Activator Ratio** | 2 | 10 |
| $\boldsymbol{p_{propene}}$ | 95 psi (6.6 bar) | 115 psi (7.9 bar) |
| **Solvent (cell, chaser and buffer)** | toluene | mixed alkane diluent (ISOPAR-G) |

The system is then thermostated at the desired polymerization temperature and brought to the desired of pressure with propene (Table S1). At this point, the catalyst injection sequence is started; aliquots of (a) a solvent 'chaser', (b) a toluene solution of catalyst (variable amount, see Table S2), (c) a toluene solution of the AB or TTB activator (variable ratio, see Table S2), and (d) a solvent 'buffer', all separated by nitrogen gaps, are uploaded into the needle and subsequently injected into the cell of destination in reverse order, thus starting the reaction. The pre-catalysts were injected into the PPR cells without pre-activation. The polymerization is left to proceed under stirring (800 rpm) at constant temperature and pressure with feed of propene on demand until the desired monomer consumption has been reached (for reaction time, see Table S2), and quenched by over-pressurizing the cell with 50 psi (3.4 bar) of dry air (preferred over other possible catalyst quenchers because in case of cell or quench line leakage oxygen is promptly detected by the dedicated glove-box sensor).

## 2.2 Polymer workup (PPR)

Once all cells have been quenched, the modules are cooled down to glovebox temperature and vented, the stir-tops are removed, and the glass inserts containing the reaction phases are taken out and transferred to a centrifugal evaporator (Genevac EZ-2 Plus or Martin Christ RVC 2-33 CDplus), where all volatiles are removed, and the polymers are thoroughly dried overnight under vacuum. Reaction yields are double-checked against on-line monomer conversion measurements by robotically weighing the dry polymers while still in the reaction vials, subtracting the pre-recorded tare. Polymer aliquots are then sent to the characterizations.

## 2.3 Polymer analytical characterizations

All polymers were characterized by means of high-temperature gel permeation chromatography (GPC) and $^{13}$C NMR spectroscopy. Chain end analysis with $^{1}$H NMR was run on selected samples. GPC curves were recorded with a Freeslate Rapid GPC setup, equipped with a set of two mixed-bed Agilent PLgel 10 $\mu$m columns and a Polymer Char IR4 detector using *ortho*-dichlorobenzene (with BHT added as a stabilizer, [BHT] = 0.4 mg mL$^{-1}$). Calibration was performed with the universal method, using 10 monodisperse polystyrene samples ($M_n$ between 1.3 and 3700 kDa).

Quantitative spectra were recorded using a Bruker Avance III 400 spectrometer equipped with a high-temperature cryoprobe for 5 mm OD tubes, on 45 mg mL$^{-1}$ polymer solutions in tetrachloroethane-1,2-$d_2$ (with BHT added as a stabilizer, [BHT] = 0.4 mg mL$^{-1}$). Acquisition conditions for $^{13}$C NMR were: 45° pulse; acquisition time, 2.7 s; relaxation delay, 3.3 s; 1-10 K transients. Broad-band proton decoupling was achieved with a modified WALTZ16 sequence (BI_WALTZ16_32 by Bruker). Acquisition conditions for $^{1}$H NMR were: 90° pulse; acquisition time, 2.0 s; relaxation delay, 10 s; 16 transients.

**Table S2.** Propene Polymerization Experiments (PPR).

| Catalyst | *T* (°C) | *p* (psi) | Scavenger/ Activator | B/Zr ratio | Yield (mg) | Cat (nmol) | *t* (s) | $R_p$[a] | | $M_n$ (kDa) | | $M_w$ (kDa) | | PDI | | 1-$\sigma$[b] | | *[2,1]*[c] | *[3,1]*[c] | $regio_{tot}$[d] | |
|---|---|---|---|---|---|---|---|---|---|---|---|---|---|---|---|---|---|---|---|---|---|
| | | | | | | | | | | | Average | | Average | | Average | | Average | | | | Average |
| | | | | 10 | 32 | 20 | 484 | 12 | | 345 | | 757 | | 2.2 | | 0.15 | | 0.07 | 0.02 | 0.09 | |
| | | | | 10 | 86 | 20 | 552 | 28 | 25 | 392 | 385 | 815 | 785 | 2.1 | 2.0 | 0.13 | | 0.06 | 0.015 | 0.075 | |
| | | | TIBA/TTB | 10 | 103 | 20 | 539 | 34 | | 434 | | 793 | | 1.8 | | 0.13 | 0.14 | 0.06 | 0.20 | 0.08 | |
| **Hf-1** | 60 | 95 | | 10 | 83 | 20 | 568 | 26 | | 370 | | 775 | | 2.1 | | 0.14 | | 0.05 | 0.02 | 0.07 | 0.08 |
| | | | TIBA/AB | 2 | 56 | 20 | 2724 | 4 | 4 | 379 | 379 | 978 | 978 | 2.6 | 2.6 | 0.14 | | 0.06 | 0.02 | 0.08 | |
| | | | | 10 | 70 | 40 | 299 | 21 | | 32 | | 64 | | 2.0 | | 0.67 | | - | 0.09 | 0.09 | |
| | | | TIBA/BHT/TTB | 10 | 78 | 30 | 254 | 37 | 20 | 27 | 31 | 53 | 62 | 2.0 | 2.0 | 0.67 | 0.67 | 0.03 | 0.11 | 0.14 | |
| | | | | 10 | 26 | 30 | 1613 | 2 | | 33 | | 70 | | 2.1 | | 0.67 | | 0.03 | 0.11 | 0.14 | |
| | | | | 2 | 72 | 20 | 1466 | 9 | | 192 | | 425 | | 2.2 | | 0.11 | | 0.14 | 0.08 | 0.22 | |
| | 60 | 95 | TIBA/AB | 2 | 23 | 40 | 5402 | 0 | 3 | 220 | 210 | 553 | 496 | 2.5 | 2.4 | 0.10 | 0.10 | 0.15 | 0.09 | 0.24 | 0.24 |
| **Hf-2** | | | | 2 | 25 | 40 | 3602 | 1 | | 217 | | 509 | | 2.3 | | 0.10 | | 0.16 | 0.10 | 0.26 | |
| | 100 | 115 | TIBA/BHT/TTB | 10 | 33 | 30 | 413 | 10 | 68 | 17 | 16 | 33 | 31 | 1.9 | 2.0 | 0.69 | 0.75 | 0.04 | 0.24 | 0.28 | 0.28 |
| | | | | 10 | 112 | 30 | 106 | 127 | | 14 | | 28 | | 2.0 | | 0.81 | | 0.03 | 0.25 | 0.28 | |
| | | | | 2 | 28 | 60 | 1774 | 1 | | 1336 | | 2886 | | 2.2 | | 0.15 | | 0.07 | n.d. | 0.07 | |
| | 60 | 95 | TIBA/AB | 2 | 53 | 60 | 3495 | 1 | 1 | 1691 | 1454 | 3485 | 3198 | 2.1 | 2.2 | 0.19 | 0.17 | 0.07 | n.d | 0.07 | 0.07 |
| | | | | 2 | 67 | 60 | 3179 | 1 | | 1336 | | 3222 | | 2.4 | | 0.17 | | 0.07 | n.d | 0.07 | |
| **Hf-3** | | | TIBA/BHT/TTB | 10 | 68 | 80 | 438 | 7 | 5 | 67 | 59 | 120 | 117 | 1.8 | 2.0 | 0.30 | | 0.04 | 0.125 | 0.165 | |
| | 100 | 115 | | 10 | 49 | 80 | 892 | 2 | | 51 | | 114 | | 2.2 | | 0.29 | 0.30 | 0.04 | 0.135 | 0.175 | 0.17 |
| | | | TIBA/TTB | 10 | 74 | 20 | 160 | 83 | 52 | 56 | 62 | 114 | 126 | 2.0 | 2.0 | 0.30 | | 0.04 | 0.13 | 0.17 | |
| | | | | 10 | 28 | 10 | 467 | 22 | | 67 | | 138 | | 2.1 | | 0.30 | | 0.04 | 0.135 | 0.175 | |
| | | | | 10 | 70 | 20 | 1492 | 8 | | 1704 | | 4269 | | 2.5 | | 0.06 | | 0.41 | 0.13 | 0.54 | |
| | | | TIBA/BHT/TTB | 10 | 46 | 20 | 1778 | 5 | 7 | 2396 | 1933 | 4778 | 4150 | 2.0 | 2.2 | 0.07 | | 0.36 | 0.12 | 0.48 | |
| | 60 | 95 | | 10 | 80 | 20 | 2184 | 7 | | 1698 | | 3402 | | 2.0 | | 0.08 | 0.07 | 0.39 | 0.11 | 0.50 | 0.49 |
| | | | TIBA/TTB | 2 | 93 | 20 | 765 | 22 | 22 | 1177 | 1159 | 2889 | 2772 | 2.5 | 2.4 | 0.07 | | 0.38 | 0.11 | 0.49 | |
| **Hf-4** | | | | 10 | 82 | 30 | 618 | 16 | 16 | 1140 | | 2655 | | 2.3 | | 0.09 | | 0.35 | 0.11 | 0.46 | |
| | 100 | 115 | TIBA/TTB/BHT | 10 | 61 | 30 | 455 | 16 | 14 | 113 | 120 | 207 | 225 | 1.8 | 1.9 | 0.21 | | 0.12 | 0.41 | 0.53 | |
| | | | | 10 | 52 | 30 | 502 | 12 | | 127 | | 243 | | 1.9 | | 0.21 | 0.21 | 0.14 | 0.44 | 0.58 | 0.56 |
| | 100 | 115 | TIBA/TTB | 10 | 20 | 20 | 2786 | 1 | 3 | 149 | 135 | 273 | 263 | 1.8 | 2.0 | 0.21 | | 0.13 | 0.43 | 0.56 | |
| | | | | 10 | 17 | 20 | 569 | 5 | | 120 | | 252 | | 2.1 | | 0.21 | | 0.14 | 0.41 | 0.55 | |
| | | | | | 50 | 3 | 1357 | 44 | 44 | 1454 | | 2997 | | 2.1 | | 0.03 | | 0.10 | n.d. | 0.10 | |
| | 60 | 95 | TIBA/BHT/AB | 2 | 35 | 3 | 2050 | 20 | 28 | 1556 | 1440 | 3531 | 3246 | 2.3 | 2.3 | <0.02 | 0.03 | 0.12 | n.d. | 0.12 | 0.11 |
| | | | | | 29 | 3 | 977 | 36 | | 1310 | | 3211 | | 2.5 | | 0.03 | | 0.11 | n.d. | 0.11 | |
| | | | TIBA/BHT/AB | 10 | 44 | 5 | 605 | 52 | 59 | 79 | 79 | 171 | 164 | 2.2 | 2.1 | 0.087 | | 0.12 | 0.06 | 0.18 | |
| **Hf-5** | | | | | 45 | 5 | 501 | 65 | | 78 | | 157 | | 2.0 | | 0.09 | | 0.11 | 0.07 | 0.18 | |
| | | | TIBA/BHT/TTB | 10 | 38 | 5 | 747 | 37 | 37 | 108 | 108 | 220 | 220 | 2.0 | 2.0 | 0.10 | | 0.13 | 0.06 | 0.19 | |
| | 100 | 115 | | | 25 | 3 | 820 | 37 | | 89 | | 174 | | 2.0 | | 0.10 | 0.10 | 0.12 | 0.06 | 0.18 | 0.18 |
| | | | TIBA/TTB | 10 | 38 | 5 | 883 | 31 | 44 | 103 | 90 | 186 | 179 | 1.8 | 2.0 | 0.11 | | 0.11 | 0.07 | 0.18 | |
| | | | | | 49 | 5 | 464 | 76 | | 90 | | 183 | | 2.0 | | 0.085 | | 0.14 | 0.06 | 0.20 | |
| | | | | | 30 | | 679 | 32 | | 78 | | 173 | | 2.2 | | 0.10 | | 0.11 | 0.06 | 0.17 | |

[a] in kg $mmol_{Zr}^{-1}$ $h^{-1}$, [b] Fraction of stereoirregular monomeric units in %, according to the enantiomorphic-site statistical model [c] $^{13}C$ NMR mole fractions of 2,1 and 3,1 monomeric units, [d] sum of 2,1 and 3,1 regioerrors

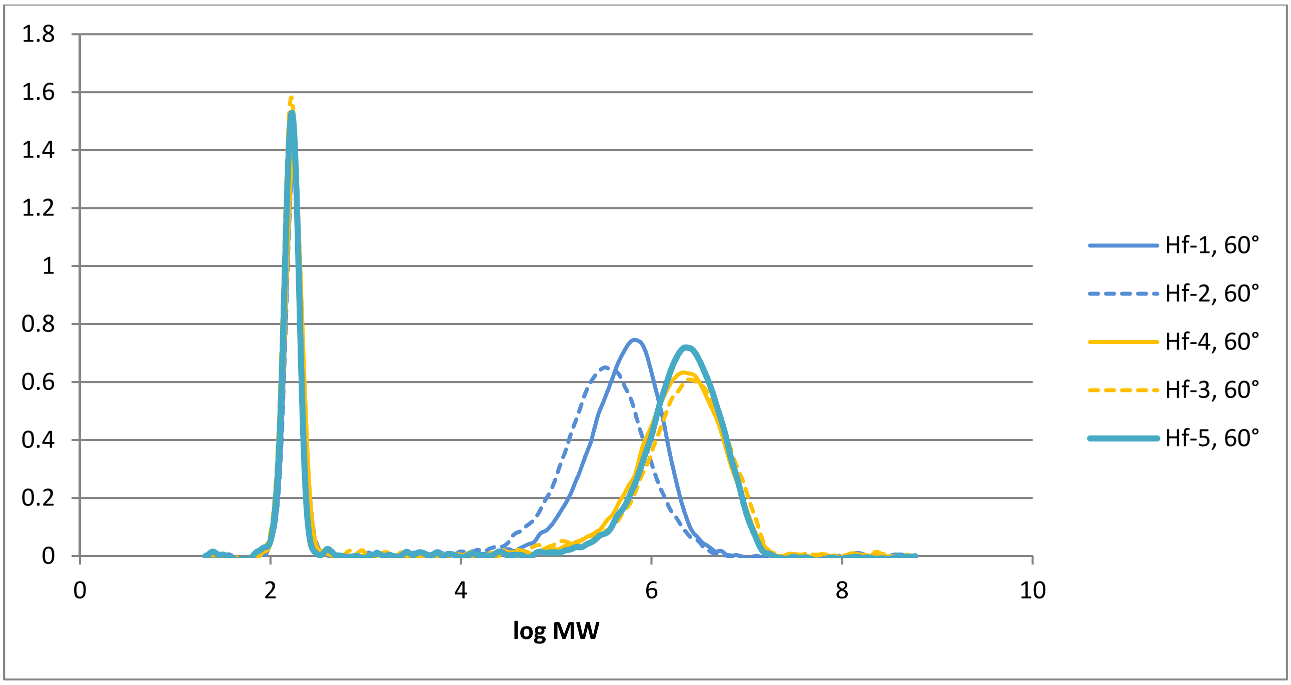


**Figure S1.** Exemplary Rapid-GPC curves for i-PP samples produced with catalysts Hf-1 to Hf-2 at 60°C (the sharp peak at log MW ~2 is due to the stabilizer).

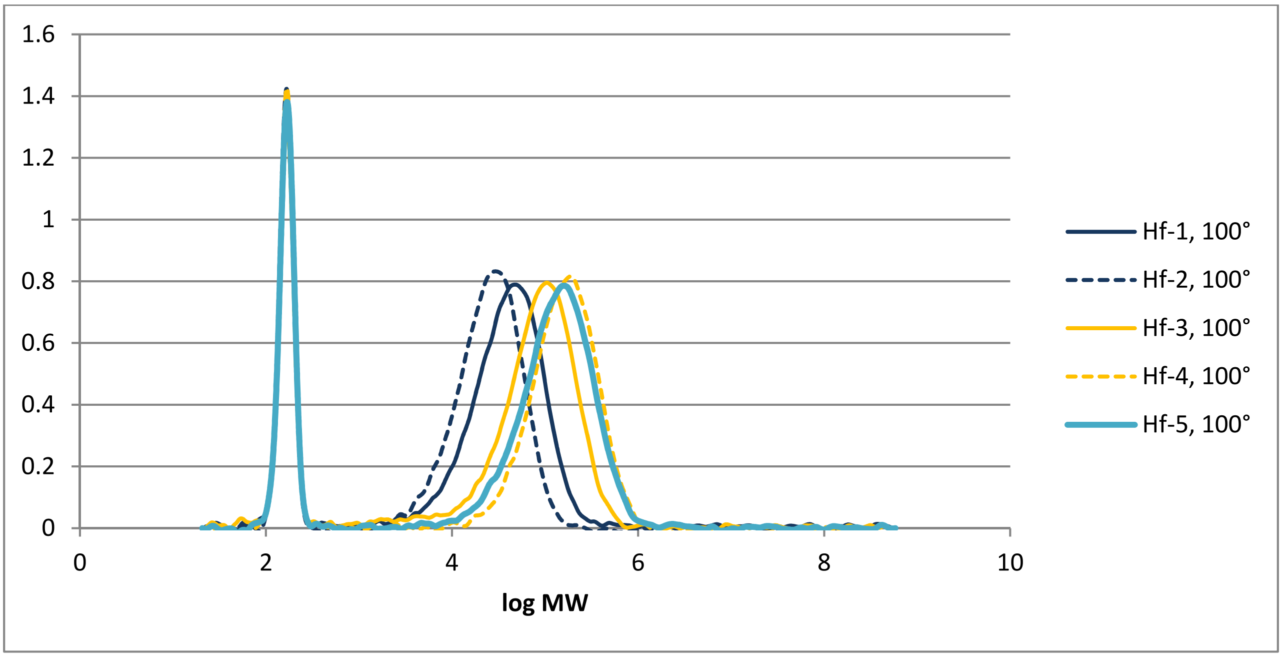


**Figure S2.** Exemplary Rapid-GPC curves for i-PP samples produced with catalysts Hf-1 to Hf-2 at 100°C (the sharp peak at log MW ~2 is due to the stabilizer).

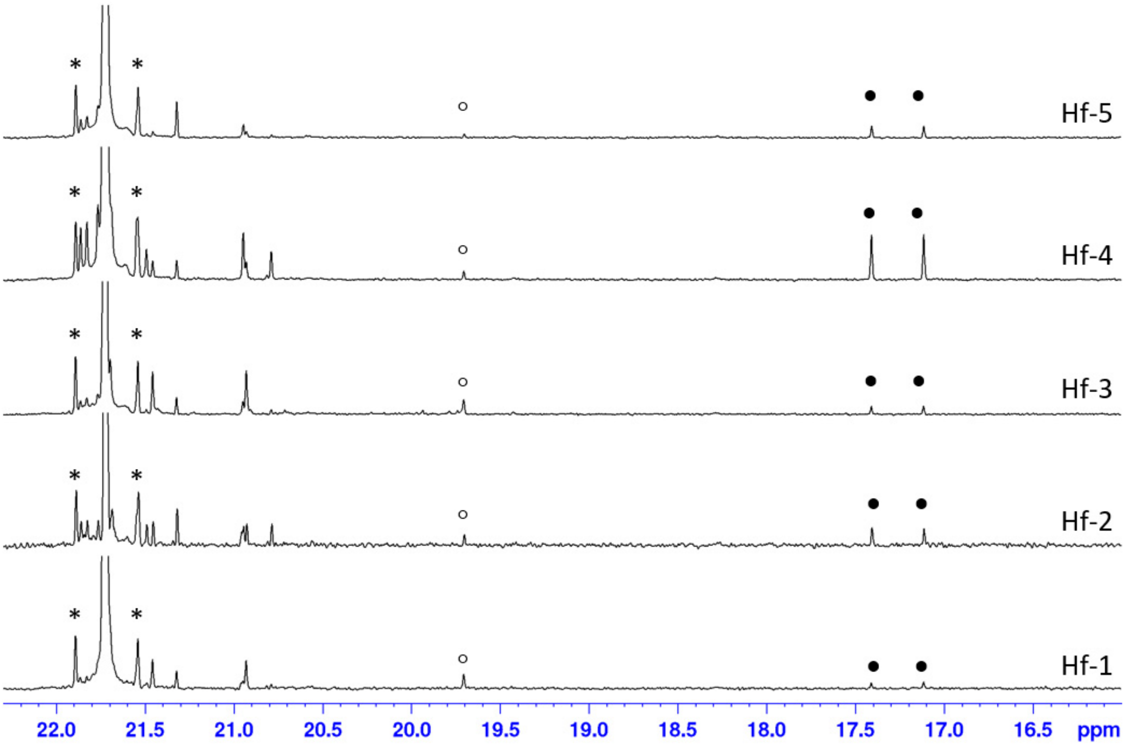


**Figure S3**. $^{13}C$ NMR spectra (expanded in the methyl region) of some representative samples obtained with catalysts Hf-1 to Hf-5 at 60°C (peaks marked with *, ◦ and • are due to satellite bands, *mmrrmm* heptad and 2,1 units, respectively).

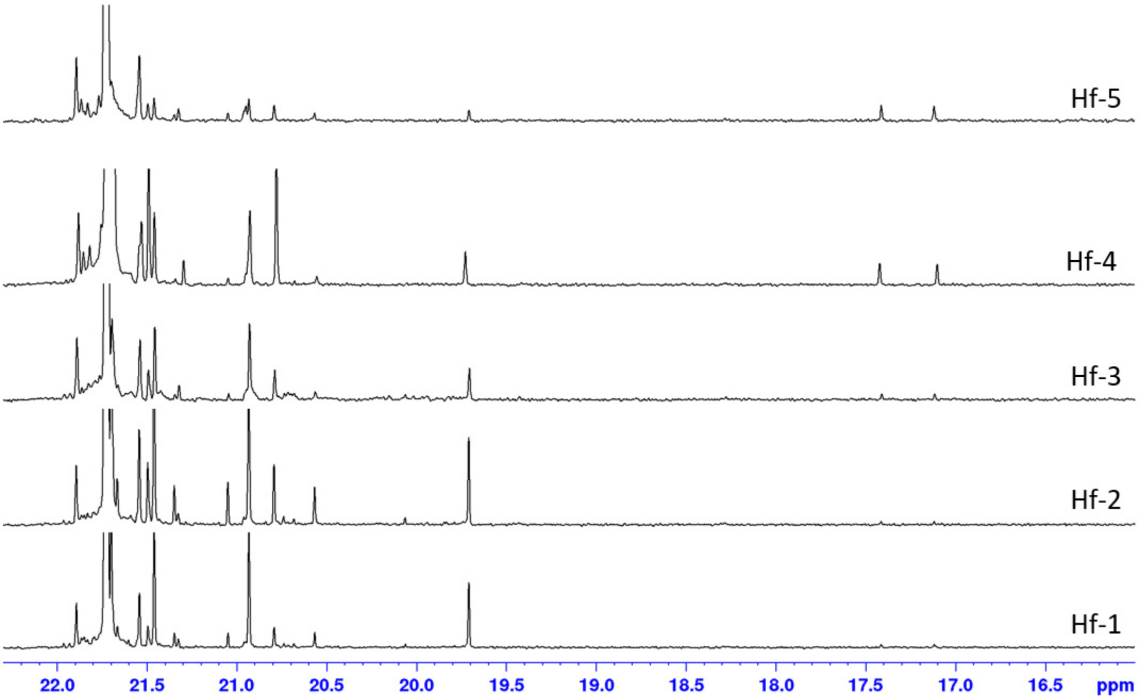


**Figure S4**. $^{13}C$ NMR spectra (expanded in the methyl region) of some representative samples obtained with catalysts Hf-1 to Hf-5 at 100°C.

# 3. QSAR Modeling

## 3.1 QSAR Modeling – Equations for Stereo-, Regioselectivity and Molecular Weight Capability Models

The following QSAR equations, previously developed for zirconocenes,[5] were used to predict the performance of catalysts **M-1** to **M-5** at

$T_p$ = 60°C:

**Stereoselectivity Model**

$\Delta\Delta G^{\ddagger}_{enantio, exp} = 0.474\ \Delta\%V_{Bur, Zr} - 3.247$ (Equation S1)

**Molecular Weight Model**

$\Delta\Delta G^{\ddagger}_{T, exp} = 0.127\ \Delta\%V_{Bur, Zr} + 0.023\ \%V_{Bur, C4} + 0.039\ \%V_{Bur, C5\text{-}6} - 0.220\ \%V_{Bur, open} - 0.099\ \%V_{Bur, C2\text{-}3\text{-}Front} + 13.971$ (Equation S2)

**Regioselectivity Model**

$\Delta\Delta G^{\ddagger}_{regiotot, exp} = -14.267\ e_{\text{-}ZrCl2, NPA} + 0.043\ \%V_{Bur, C2\text{-}3\text{-}All} + 0.031\ \%V_{Bur, C4} - 0.031\ \%V_{Bur, C5\text{-}6} - 0.529\ \%V_{Bur, open} + 23.619$ (Equation S3)

$T_p$ = 100°C:

**Stereoselectivity Model**

$\Delta\Delta G^{\ddagger}_{enantio, exp} = -21.469 \times q^{-}_{ZrCl2, NPA} + 0.499\ \Delta\%V_{Bur, Zr} - 3.894$ (Equation S4)

**Regioselectivity Model**

$\Delta\Delta G^{\ddagger}_{regiotot, exp} = 0.016\ \%V_{Bur, C4} - 0.030\ \%V_{Bur(C5+C6)} + 0.065\ \%V_{Bur(C2+3)} - 0.682\ \%V_{Bur, open} - 14.346\ q^{-}_{ZrCl2, NPA} + 26.820$ (Equation S5)

**Molecular Weight Model**

$\Delta\Delta G^{\ddagger}_{T, exp} = 0.157\ \Delta\%V_{Bur, Zr} + 0.030\ \%V_{Bur, C4} + 0.049\ \%V_{Bur(C5+C6)} - 0.178\ \%V_{Bur(C2+C3),Front} + 7.967$ (Equation S6)

The descriptor values were determined as described in Ref. 5, except for $\%V_{Bur}$ for **Hf-5**, which is used to screen steric bulk coming backwards from the active pocket. In this case, the triptycene part linked to the 5-position (going away from the active pocket) was additionally deleted.[6]

## 3.2 Predicted vs. observed performance for **Hf-1 to Hf-5**

**Table S3.** Predicted vs. observed performance in terms of stereoselectivity (Equation S1), regioselectivity (Equation S2) and molecular weight capability (Equation S3) of **Hf-1 to Hf-5** (QSAR/Experimental values) at 60°C.

| **Catalyst** | **1-$\sigma$ (%)** | ***regio*$_{tot}$ (%)** | **$M_n$ (kDa)** |
|---|---|---|---|
| **Hf-1** | 0.08/0.14 | 0.29/0.08 | 380/390 |
| **Hf-2** | 0.04/0.10 | 0.63/0.24 | 330/210 |
| **Hf-3** | 0.10/0.17 | 0.33/0.07 | 270/1500 |
| **Hf-4** | 0.03/0.07 | 1.32/0.50 | 1200/1900 |
| **Hf-5** | 0.01/0.03 | 0.19/0.11 | 2700/1400 |

| **Catalyst** | **$\Delta\Delta G^{‡}_{enantio}$ (kcal/mol)** | **$\Delta\Delta G^{‡}_{regio}$ (kcal/mol)** | **$\Delta\Delta G^{‡}_{T}$ (kcal/mol)** |
|---|---|---|---|
| **Hf-1** | 4.8/4.4 | 3.9/4.8 | 6.0/6.1 |
| **Hf-2** | 5.2/4.6 | 3.4/4.0 | 5.9/5.6 |
| **Hf-3** | 4.6/4.2 | 3.8/4.8 | 5.8/6.9 |
| **Hf-4** | 5.5/4.8 | 2.9/3.5 | 6.8/7.1 |
| **Hf-5** | 6.2/5.4 | 4.1/4.5 | 7.3/6.9 |

**Table S4.** Predicted vs. observed performance in terms of stereoselectivity (Equation S4), regioselectivity (Equation S5) and molecular weight capability (Equation S6) of **Hf-1 to Hf-5** (QSAR/Experimental values) at 100°C.

| **Catalyst** | **1-$\sigma$ (%)** | ***regio*$_{tot}$ (%)** | **$M_n$ (kDa)** |
|---|---|---|---|
| **Hf-1** | 0.25/0.63 | 0.64/0.12 | 47/31 |
| **Hf-2** | 0.16/0.72 | 1.06/0.28 | 40/16 |
| **Hf-3** | 0.25/0.30 | 0.76/0.17 | 41/60 |
| **Hf-4** | 0.07/0.21 | 2.02/0.56 | 157/140 |
| **Hf-5** | 0.02/0.10 | 0.57/0.18 | 372/90 |

| **Catalyst** | **$\Delta\Delta G^{‡}_{enantio}$ (kcal/mol)** | **$\Delta\Delta G^{‡}_{regio}$ (kcal/mol)** | **$\Delta\Delta G^{‡}_{T}$ (kcal/mol)** |
|---|---|---|---|
| **Hf-1** | 4.4/3.8 | 3.7/5.0 | 5.2/4.9 |
| **Hf-2** | 4.8/3.7 | 3.4/4.4 | 5.1/4.4 |
| **Hf-3** | 4.4/4.3 | 3.6/4.7 | 5.1/5.4 |
| **Hf-4** | 5.4/4.6 | 2.9/3.8 | 6.1/6.0 |
| **Hf-5** | 6.3/5.1 | 3.8/4.7 | 6.7/5.7 |

## 3.3 QSAR Models for Chain End Termination and Propensity for 1,2-to-1,3 regioerror Isomerization

### 3.3.1 Chain End Termination Model

**Table S5.** Computational descriptors, experimental performance indicator, and experimental and QSAR predicted $\Delta G^{\ddagger}_{BME\text{-}BHET}$ (using Hirschfeld or NPA chargers).

| | Computational descriptors | | | Chain ends analysis | | | $\Delta G^{\ddagger}_{BME\text{-}BHET}$ | | |
|---|---|---|---|---|---|---|---|---|---|
| **ID** | $q^{-}_{ZrCl_2,NPA}$ | $q^{-}_{ZrCl_2,HF}$ | $\Delta\%V_{bur(5.0)}$ | **Vinylidene** | **Ally** | **Vd/allyl** | **EXP** | **QSAR (Hirschfeld)** | **QSAR (NPA)** |
| **Zr-1** | 0.408 | 0.0155 | 16.8 | 0.220 | 0.220 | 1.0 | 0.0 | -0.39 | 0.18 |
| **Zr-2** | 0.414 | 0.0272 | 17.6 | 0.050 | 0.060 | 1.2 | 0.1 | 0.23 | 1.03 |
| **Zr-3** | 0.397 | 0.0074 | 16.2 | 0.062 | 0.013 | 0.2 | -1.2 | -0.83 | -0.85 |
| **Zr-4** | 0.400 | 0.0211 | 17.6 | 0.007 | 0.007 | 1.0 | 0.0 | 0.05 | 0.16 |
| **Zr-5** | 0.396 | 0.0142 | 19.2 | 0.007 | 0.013 | 1.9 | 0.5 | 0.38 | 0.85 |
| **Hf-1** | 0.414 | 0.0594 | 16.9 | 0.020 | 0.105 | 5.3 | 1.2 | 0.95 | 0.61 |
| **Hf-2** | 0.421 | 0.0692 | 17.9 | 0.032 | 0.240 | 7.5 | 1.5 | 1.59 | 1.64 |
| **Hf-3** | 0.405 | 0.0497 | 16.5 | 0.042 | 0.081 | 1.9 | 0.5 | 0.53 | -0.18 |
| **Hf-4** | 0.406 | 0.0632 | 18.4 | 0.005 | 0.034 | 6.8 | 1.4 | 1.58 | 1.00 |
| **Hf-5** | 0.400 | 0.0573 | 20.0 | 0.003 | 0.042 | 14.0 | 2.0 | 1.94 | 1.57 |

SUMMARY OUTPUT

| *Regression Statistics* | |
|---|---|
| Multiple R | 0.975008 |
| R Square | 0.950641 |
| Adjusted F | 0.936538 |
| Standard E | 0.233941 |
| Observatic | 10 |

ANOVA

| | *df* | *SS* | *MS* | *F* | *Significance F* |
|---|---|---|---|---|---|
| Regressior | 2 | 7.378345 | 3.689173 | 67.40902954 | 2.67167E-05 |
| Residual | 7 | 0.383097 | 0.054728 | | |
| Total | 9 | 7.761442 | | | |

| | *Coefficients* | *andard Err* | *t Stat* | *P-value* | *Lower 95%* | *Upper 95%* | *Lower 95.0%* | *Upper 95.0%* |
|---|---|---|---|---|---|---|---|---|
| Intercept | -6.53691 | 1.157497 | -5.64745 | 0.000776536 | -9.27395263 | -3.79986296 | -9.27395263 | -3.79986296 |
| X Variable | 29.85651 | 3.444811 | 8.667099 | 5.44713E-05 | 21.71083209 | 38.00219727 | 21.71083209 | 38.00219727 |
| X Variable | 0.338334 | 0.066873 | 5.059355 | 0.001464409 | 0.180204396 | 0.496462788 | 0.180204396 | 0.496462788 |

**Figure S5.** Analysis of variance, chain end termination model

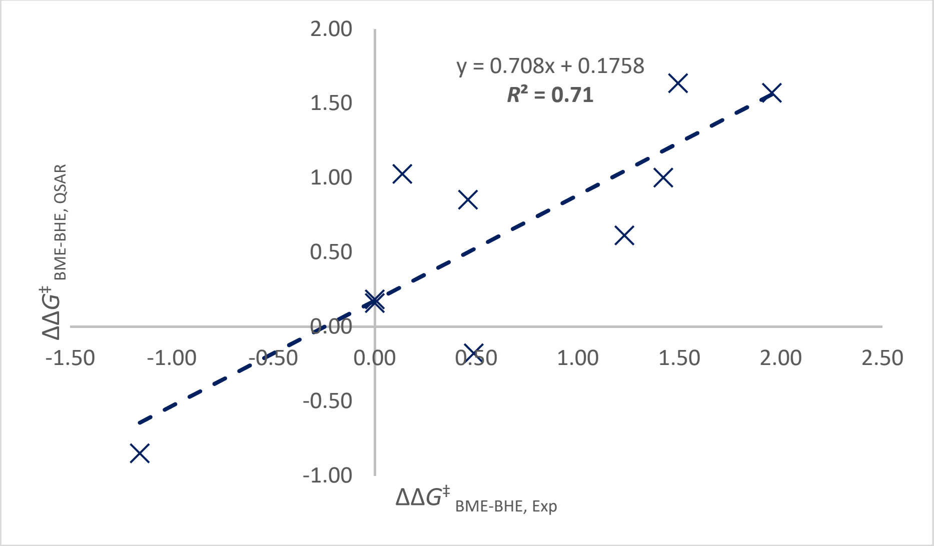


**Figure S6.** Correlation of experimentally observed ratio of allyl to vinyl chain ends (modeled as $\Delta\Delta G^{\ddagger}_{\text{BME-BHET, Exp}}$) at 100°C and QSAR predicted ratio ($\Delta\Delta G^{\ddagger}_{\text{BME-BHET, QSAR}}$) for catalysts **Hf-1** to **Hf-5** and **Zr-1** to **Zr-5**, using NPA rather than Hirschfeld charges.

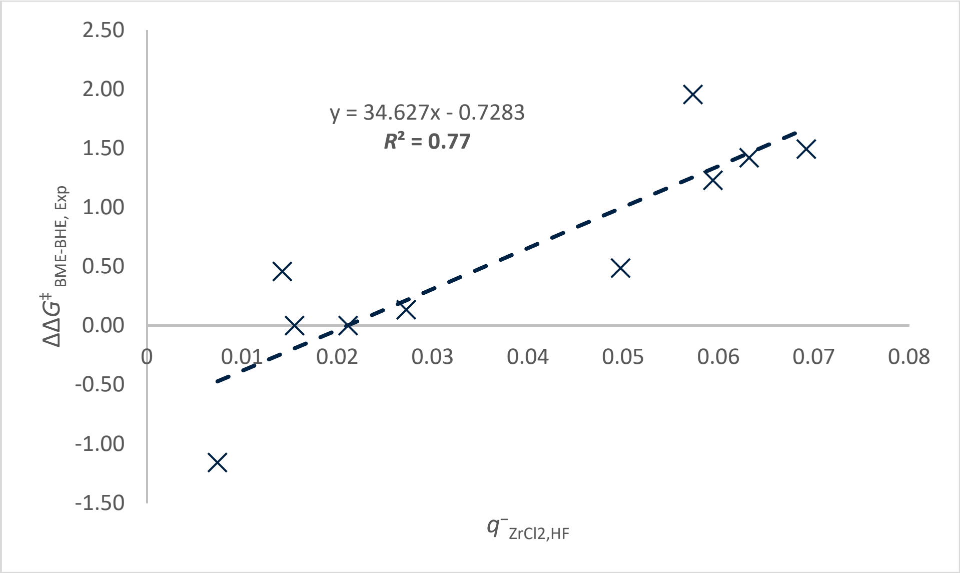


**Figure S7.** Single descriptor correlation of $q^{-}_{\text{ZrCl}_2\text{,HF}}$ with $\Delta G^{\ddagger}_{\text{BME-BHE}_\text{T}}$.

### 3.3.2 Model for Propensity for 1,2-to-1,3 regioerror Isomerization

**Table S6.** Computational descriptors, experimental performance indicator, and experimental and QSAR predicted $\Delta\Delta G^{‡}_{1,2\text{-}2,1\text{INS-BHE}}$ (using Hirschfeld chargers).

| | **Computational descriptors** | | **Regioerrors** | | | **$\Delta\Delta G^{‡}_{1,2\text{-}2,1\text{INS-BHE}}$** | |
|---|---|---|---|---|---|---|---|
| **ID** | **$q^{-}_{ZrCl_2,HF}$** | **$\Delta\%V_{bur(5.0)}$** | **2,1** | **3,1** | **2,1/3,1** | **EXP** | **QSAR** |
| **Zr-1** | 0.0155 | 16.8 | 0.21 | 0.09 | 2.3 | 0.6 | 0.397 |
| **Zr-2** | 0.0272 | 17.6 | 0.19 | 0.33 | 0.6 | -0.4 | 0.334 |
| **Zr-3** | 0.0074 | 16.2 | 0.29 | 0.14 | 2.1 | 0.5 | 0.417 |
| **Zr-4** | 0.0211 | 17.6 | 0.57 | 0.42 | 1.4 | 0.2 | 0.587 |
| **Zr-5** | 0.0142 | 19.2 | 0.33 | 0.02 | 16.5 | 2.1 | 1.716 |
| **Hf-1** | 0.0594 | 16.9 | 0.02 | 0.1 | 0.2 | -1.2 | -1.371 |
| **Hf-2** | 0.0692 | 17.9 | 0.04 | 0.25 | 0.2 | -1.4 | -1.250 |
| **Hf-3** | 0.0497 | 16.5 | 0.04 | 0.13 | 0.3 | -0.9 | -1.179 |
| **Hf-4** | 0.0632 | 18.4 | 0.13 | 0.43 | 0.3 | -0.9 | -0.737 |
| **Hf-5** | 0.0573 | 20.0 | 0.12 | 0.06 | 2.0 | 0.5 | 0.351 |

SUMMARY OUTPUT

| *Regression Statistics* | |
|---|---|
| Multiple R | 0.946543431 |
| R Square | 0.895944467 |
| Adjusted R Square | 0.866214315 |
| Standard Error | 0.390222756 |
| Observations | 10 |

ANOVA

| | *df* | *SS* | *MS* | *F* | *gnificance F* |
|---|---|---|---|---|---|
| Regression | 2 | 9.177812 | 4.588906 | 30.13589 | 0.000363 |
| Residual | 7 | 1.065917 | 0.152274 | | |
| Total | 9 | 10.24373 | | | |

| | *Coefficients* | *andard Err* | *t Stat* | *P-value* | *ower 95%* | *Upper 95%* | *Lower 95.0%* | *Upper 95.0%* |
|---|---|---|---|---|---|---|---|---|
| Intercept | -7.815715438 | 1.930754 | -4.04801 | 0.004883 | -12.3812 | -3.250208013 | -12.38122286 | -3.250208013 |
| X Variable 1 | -41.47359499 | 5.746091 | -7.21771 | 0.000175 | -55.0609 | -27.8862493 | -55.06094069 | -27.8862493 |
| X Variable 2 | 0.527140359 | 0.111547 | 4.725733 | 0.002143 | 0.263374 | 0.790906597 | 0.263374121 | 0.790906597 |

**Figure S8.** Analysis of variance, 1,2-to-1,3 regioerror isomerization Model.

## 3.4 Final Energies, Enthalpies and Free Energies for species in Figure 5.

**Table S7.** Final energies, entropy and enthalpy corrections (T=273, 333 or 373 K, p=1.0 atm) in Hartree.

| Structure | Formula | Energy(MN15/DZ) | Energy(MN15/TZ) | Num Negative | ZPE() | EnthalpyCorr (t=273, p=1.0) | EntropyCorr (t=273, p=1.0) | EnthalpyCorr (t=333, p=1.0) | EntropyCorr (t=333, p=1.0) | EnthalpyCorr (t=373, p=1.0) | EntropyCorr (t=373, p=1.0) | E | H(273) | G(273) | H(333) | G(333) | H(373) | G(373) |
|---|---|---|---|---|---|---|---|---|---|---|---|---|---|---|---|---|---|---|
| propene | C3H6 | -117.7392684 | -117.7871194 | 0 | 0.07955 | 0.084011159 | 0.026957087 | 0.085478331 | 0.034495314 | 0.086584344 | 0.039807796 | -117.70757 | -117.703108 | -117.721169 | -117.701641 | -117.724753 | -117.700535 | -117.7272062 |
| isobutene | C4H8 | -157.0039828 | -157.0669327 | 0 | 0.105299 | 0.111431588 | 0.031802029 | 0.113506645 | 0.041073986 | 0.11505905 | 0.047648562 | -156.961634 | -156.955501 | -156.976808 | -156.953426 | -156.980946 | -156.951874 | -156.9837982 |
| | | | | | | | | | | | | | | | | | | |
| **Zr** | | | | | | | | | | | | | | | | | | |
| PP_RS_iBu_beta+ | C40H43SiZr | -1884.431039 | -1884.971341 | 0 | 0.709408 | 0.745385088 | 0.096752612 | 0.761740042 | 0.135996475 | 0.774248321 | 0.165551035 | -1884.26193 | -1884.22596 | -1884.29078 | -1884.2096 | -1884.30072 | -1884.19709 | -1884.308011 |
| PP_TS_BHE_iBu_+ | C40H43SiZr | -1884.409965 | -1884.94699 | 1 | 0.704801 | 0.740888406 | 0.097895568 | 0.757236671 | 0.137383324 | 0.769737576 | 0.167096702 | -1884.24219 | -1884.2061 | -1884.27169 | -1884.18975 | -1884.2818 | -1884.17725 | -1884.289207 |
| PP_RS_H_isobutene+ | C40H43SiZr | -1884.426276 | -1884.960786 | 0 | 0.705868 | 0.742125198 | 0.096952923 | 0.758580632 | 0.136351603 | 0.771155158 | 0.166018908 | -1884.25492 | -1884.21866 | -1884.28362 | -1884.20221 | -1884.29356 | -1884.18963 | -1884.300863 |
| PP_RS_H_isobutene_rotated+ | C40H43SiZr | -1884.423495 | -1884.957474 | 0 | 0.705833 | 0.742277129 | 0.097638963 | 0.758765621 | 0.137224902 | 0.7713609 | 0.167019073 | -1884.25164 | -1884.2152 | -1884.28062 | -1884.19871 | -1884.29065 | -1884.18611 | -1884.298016 |
| PP_TS_BHE_tBu_+ | C40H43SiZr | -1884.413604 | -1884.94204 | 1 | 0.705095 | 0.740846521 | 0.096513905 | 0.757125164 | 0.135621347 | 0.769577043 | 0.165071229 | -1884.23695 | -1884.20119 | -1884.26586 | -1884.18492 | -1884.27578 | -1884.17246 | -1884.283061 |
| PP_RS_CMe3+ | C40H43SiZr | -1884.413303 | -1884.952594 | 0 | 0.709214 | 0.745390064 | 0.097425168 | 0.76180049 | 0.136878361 | 0.774335407 | 0.166567109 | -1884.24338 | -1884.2072 | -1884.27248 | -1884.19079 | -1884.2825 | -1884.17826 | -1884.289859 |
| PP_TS_BME_iBu+ | C40H43SiZr | -1884.410161 | -1884.943244 | 1 | 0.707654 | 0.743310018 | 0.09605638 | 0.759618063 | 0.135095335 | 0.772097332 | 0.164510968 | -1884.23559 | -1884.19993 | -1884.26429 | -1884.18363 | -1884.27414 | -1884.17115 | -1884.281369 |
| PP_RS_Me_propene+ | C40H43SiZr | -1884.425758 | -1884.95997 | 0 | 0.705398 | 0.743033775 | 0.101025838 | 0.759778784 | 0.141639802 | 0.772520354 | 0.172119231 | -1884.25457 | -1884.21694 | -1884.28462 | -1884.20019 | -1884.29509 | -1884.18745 | -1884.302769 |
| | | | | | | | | | | | | | | | | | | |
| **Hf** | | | | | | | | | | | | E | H(273) | G(273) | H(333) | G(333) | H(373) | G(373) |
| PP_RS_iBu_beta+ | C40H43HfSi | -1886.147723 | -1886.688586 | 0 | 0.709116 | 0.745185688 | 0.097187261 | 0.761555814 | 0.136543436 | 0.77407268 | 0.166172791 | -1885.97947 | -1885.9434 | -1886.00852 | -1885.92703 | -1886.01851 | -1885.91451 | -1886.025849 |
| PP_TS_BHE_iBu_+ | C40H43HfSi | -1886.129288 | -1886.666777 | 1 | 0.704973 | 0.740960846 | 0.097275351 | 0.757311202 | 0.136629101 | 0.769813355 | 0.166253204 | -1885.9618 | -1885.92582 | -1885.99099 | -1885.90947 | -1886.00101 | -1885.89696 | -1886.008353 |
| PP_RS_H_isobutene+ | C40H43HfSi | -1886.143205 | -1886.681974 | 0 | 0.704393 | 0.741610315 | 0.100462252 | 0.758195755 | 0.140775947 | 0.770845934 | 0.17105481 | -1885.97758 | -1885.94036 | -1886.00767 | -1885.92378 | -1886.0181 | -1885.91113 | -1886.025734 |
| PP_RS_H_isobutene_rotated+ | C40H43HfSi | -1886.142006 | -1886.678244 | 0 | 0.705462 | 0.742092944 | 0.097973572 | 0.758618582 | 0.137674151 | 0.771234674 | 0.167544331 | -1885.97278 | -1885.93615 | -1886.00179 | -1885.91963 | -1886.01187 | -1885.90701 | -1886.019264 |
| PP_TS_BHE_tBu_+ | C40H43HfSi | -1886.128342 | -1886.658359 | 1 | 0.704361 | 0.74053797 | 0.098173385 | 0.75685948 | 0.137693084 | 0.769332759 | 0.167414508 | -1885.954 | -1885.91782 | -1885.9836 | -1885.9015 | -1885.99375 | -1885.88903 | -1886.001194 |
| PP_RS_CMe3+ | C40H43HfSi | -1886.131001 | -1886.67026 | 0 | 0.709316 | 0.74548439 | 0.097447722 | 0.761901414 | 0.136913155 | 0.774440348 | 0.166610335 | -1885.96094 | -1885.92478 | -1885.99007 | -1885.90836 | -1886.00009 | -1885.89582 | -1886.007449 |
| PP_TS_BME_iBu+ | C40H43HfSi | -1886.13051 | -1886.662533 | 1 | 0.707553 | 0.743284156 | 0.09640604 | 0.759602074 | 0.135532791 | 0.772086284 | 0.165006209 | -1885.95498 | -1885.91925 | -1885.98384 | -1885.90293 | -1885.99374 | -1885.89045 | -1886.001001 |
| PP_RS_Me_propene+ | C40H43HfSi | -1886.146179 | -1886.680682 | 0 | 0.706098 | 0.743429524 | 0.099518872 | 0.760145832 | 0.139769906 | 0.77287045 | 0.170006776 | -1885.97458 | -1885.93725 | -1886.00393 | -1885.92054 | -1886.01418 | -1885.90781 | -1886.021716 |

**Table S8.** Relative enthalpies and Gibbs free energies (T=273, 333 or 373 K, p=1.0 atm) in kcal/mol.

| Relative Enthalpies and Gibbs Free Energies | | | | | | |
|---|---|---|---|---|---|---|
| **H(273)** | **G(273)** | **H(333)** | **G(333)** | **H(373)** | **G(373)** | **Zr** |
| 0.0 | **0.0** | 0.0 | **0.0** | 0.0 | **0.0** | PP_RS_iBu_beta+ |
| 12.5 | **12.0** | 12.5 | **11.9** | 12.4 | **11.8** | PP_TS_BHE_iBu_+ |
| 4.6 | **4.5** | 4.6 | **4.5** | 4.7 | **4.5** | PP_RS_H_isobutene+ |
| 6.8 | **6.4** | 6.8 | **6.3** | 6.9 | **6.3** | PP_RS_H_isobutene_rotated+ |
| 15.5 | **15.6** | 15.5 | **15.6** | 15.5 | **15.7** | PP_TS_BHE_tBu_+ |
| 11.8 | **11.5** | 11.8 | **11.4** | 11.8 | **11.4** | PP_RS_CMe3+ |
| 16.3 | **16.6** | 16.3 | **16.7** | 16.3 | **16.7** | PP_TS_BME_iBu+ |
| 5.7 | **3.9** | 5.9 | **3.5** | 6.1 | **3.3** | PP_RS_Me_propene+ |
| | | | | | | |
| **H(273)** | **G(273)** | **H(333)** | **G(333)** | **H(373)** | **G(373)** | **Hf** |
| 0.0 | **0.0** | 0.0 | **0.0** | 0.0 | **0.0** | PP_RS_iBu_beta+ |
| 11.0 | **11.0** | 11.0 | **11.0** | 11.0 | **11.0** | PP_TS_BHE_iBu_+ |
| 1.9 | **0.5** | 2.0 | **0.3** | 2.1 | **0.1** | PP_RS_H_isobutene+ |
| 4.5 | **4.2** | 4.6 | **4.2** | 4.7 | **4.1** | PP_RS_H_isobutene_rotated+ |
| 16.1 | **15.6** | 16.0 | **15.5** | 16.0 | **15.5** | PP_TS_BHE_tBu_+ |
| 11.7 | **11.6** | 11.7 | **11.6** | 11.7 | **11.5** | PP_RS_CMe3+ |
| 15.2 | **15.5** | 15.1 | **15.5** | 15.1 | **15.6** | PP_TS_BME_iBu+ |
| 3.9 | **2.9** | 4.1 | **2.7** | 4.2 | **2.6** | PP_RS_Me_propene+ |

# 4. References


(1) Schöbel, A.; Herdtweck, E.; Parkinson, M.; Rieger, B. Ultra-Rigid Metallocenes for Highly Iso- and Regiospecific Polymerization of Propene: The Search for the Perfect Polypropylene Helix. *Chem. - Eur. J.* **2012**, *18*, 4174–4178.

(2) Busico, V.; Cipullo, R.; Mingione, A.; Rongo, L. Accelerating the Research Approach to Ziegler-Natta Catalysts. *Ind. Eng. Chem. Res.* **2016**, *55*, 2686–2695.

(3) Busico, V.; Pellecchia, R.; Cutillo, F.; Cipullo, R. High-Throughput Screening in Olefinpolymerization Catalysis: From Serendipitous Discovery towards Rational Understanding. *Macromol. Rapid Commun.* **2009**, *30*, 1697–1708.

(4) Ehm, C.; Mingione, A.; Vittoria, A.; Zaccaria, F.; Cipullo, R.; Busico, V. High-Throughput Experimentation in Olefin Polymerization Catalysis: Facing the Challenges of Miniaturization. *Ind. Eng. Chem. Res.* **2020**, *59*, 13940–13947.

(5) Ehm, C.; Vittoria, A.; Goryunov, G. P.; Izmer, V. V; Kononovich, D. S.; Kulyabin, P. S.; Di Girolamo, R.; Budzelaar, P. H. M.; Voskoboynikov, A. Z.; Busico, V.; et al. A Systematic Study of the Temperature-Induced Performance Decline of Ansa-Metallocenes for IPP. *Macromolecules* **2020**, *53*, 9325–9336.

(6) Kulyabin, P. S.; Goryunov, G. P.; Sharikov, M. I.; Izmer, V. V.; Vittoria, A.; Budzelaar, P. H. M.; Busico, V.; Voskoboynikov, A. Z.; Ehm, C.; Cipullo, R.; et al. Ansa -Zirconocene Catalysts for Isotactic-Selective Propene Polymerization at High Temperature: A Long Story Finds a Happy Ending . *J. Am. Chem. Soc.* **2021**, *143*, 7641–7647.
